# Discovery of circular transcripts of the human BCL2-like 12 (*BCL2L12*) apoptosis-related gene, using targeted nanopore sequencing, provides new insights into circular RNA biology

**DOI:** 10.1007/s10142-025-01578-1

**Published:** 2025-03-19

**Authors:** Paraskevi Karousi, Christos K. Kontos, Stavroula T. Nikou, Thomas Carell, Diamantis C. Sideris, Andreas Scorilas

**Affiliations:** 1 Department of Biochemistry and Molecular Biology, Faculty of Biology, National and Kapodistrian University of Athens, Athens, Greece; 2 Department for Chemistry, Institute for Chemical Epigenetics, Ludwig Maximilian University of Munich, Munich, Germany

**Keywords:** CircRNAs, Alternative splicing, Alternative back-splicing, Third-generation (long-read) sequencing, Colorectal cancer, Gene expression regulation

## Abstract

Circular RNAs (circRNAs) constitute an RNA type formed by back-splicing. *BCL2-like 12* (*BCL2L12*) is an apoptosis-related gene comprising 7 exons. In this study, we used targeted nanopore sequencing to identify circular *BCL2L12* transcripts in human colorectal cancer cells and investigated the effect of circRNA silencing on mRNA expression of the parental gene. In brief, nanopore sequencing following nested PCR amplification of cDNAs of *BCL2L12* circRNAs from 7 colorectal cancer cell lines unraveled 46 *BCL2L12* circRNAs, most of which described for the first time. Interestingly, 40 novel circRNAs are likely to form via back-splicing between non-canonical back-splice sites residing in highly similar regions of the primary transcripts. All back-splice junctions were validated using next-generation sequencing (NGS) after circRNA enrichment. Surprisingly, 2 novel circRNAs also comprised a poly(A) tract after *BCL2L12* exon 7; this poly(A) tract was back-spliced to exon 1, in both cases. Furthermore, the selective silencing of a *BCL2L12* circRNA resulted in a subsequent decrease of *BCL2L12* mRNA levels in HCT 116 cells, thus providing evidence of parental gene expression regulation by circRNAs. In conclusion, our study led to the discovery of many circular transcripts from a single human gene and provided new insights into circRNA biogenesis and mode of action.

**Supplementary Information:**

The online version contains supplementary material available at 10.1007/s10142-025-01578-1.

## Introduction

In eukaryotic cells, alternative splicing is a fundamental process that has been studied for years yet continues to arouse scientific interest. This process contributes to the enrichment of the functional products derived from a single gene. The most common alternative splicing mechanisms include the use of alternative exons, retained introns, and/or alternative 5′ and 3′ splice sites. Disruption of the splicing mechanisms is a hallmark of many diseases, including cancer; there are several examples of cancer-specific alternative transcripts of proto-oncogenes, tumor suppressor and apoptosis-related genes (El Marabti and Younis 2018).

Circular RNAs (circRNAs) represent a class of non-coding RNAs, initially considered as byproducts of splicing events (Cocquerelle et al. 1993; Liu et al. 2017). These RNAs are generated by a head-to-tail splicing process named back-splicing and, hence, constitute a covalently closed loop. The main roles that have been attributed to circRNAs are regulatory (Memczak et al. 2013); however, in some instances, they undergo translation. Their functions include sponging of microRNAs (miRNAs), interactions with RNA-binding proteins (RBPs), acting as scaffolds for protein complexes, and regulation of the transcription of their parental genes (Li et al. 2015; Wesselhoeft et al. 2018). To date, specific circRNA types have been described, based on whether they incorporate annotated exonic or intronic regions (Guo et al. 2014). However, recent studies have revealed circRNA sequences that are not supported by the suggested biogenesis mechanisms and which cannot be characterized as of the existing circRNA types (Rahimi et al. 2021a, b), proving that the field of circRNA biology is still in its infancy.

circRNAs have been proven to participate in the development and progression of various types of cancer, including colorectal cancer (CRC) (Artemaki et al. 2020; Papatsirou et al. 2021). CRC is a malignancy occurring in the colon or rectum, characterized by a high incidence rate in both men and women (Dekker et al. 2019). As the mortality rate of CRC is alarmingly high, scientific interest has been aroused in deciphering the molecular mechanisms underlying CRC development. Tumor-suppressor genes, oncogenes, and DNA-repair genes are heavily involved in the molecular background of CRC (Artemaki et al. 2020), while this malignancy is also characterized by the deregulation of major signaling pathways, high abundance of mutations, and chromosomal instability (De Rosa et al. 2015; Farooqi et al. 2019). As the CRC background puzzle is extremely complex, the elucidation of the involvement of apoptosis-related genes in CRC development and progression would be a wise starting point to unroll this tangle.

*BCL2L12* is a member of the BCL2 family, identified by members of our research group (Scorilas et al. 2001); it is located on chromosome 19q13.33. The main linear *BCL2L12* transcript incorporates 7 annotated exons, encoding a proline-rich protein with a BCL2-homology 2 (BH2) domain and a putative BCL2-homology 3 (BH3) domain. Hence, its structure is not typical, either for a pro- or an anti-apoptotic BCL2 family member; however, BCL2L12 is an inhibitor of CASP3 and CASP7, and also binds to P53, exerting anti-apoptotic function. Various alternative linear transcripts have been identified by members of our research group (Adamopoulos et al. 2016; Kontos and Scorilas 2012), while recently we have also discovered 2 novel *BCL2L12* circRNAs in CRC cell lines (Karousi et al. 2020). The *BCL2L12* gene is widely expressed in CRC (Mathioudaki et al. 2004), while it has also been proposed as a biomarker able to predict CRC patients’ prognosis (Kontos et al. 2008). Moreover, *BCL2L12* mRNA expression in CRC cell lines has been reported to be affected by their treatment with classical chemotherapeutic agents (Kontos et al. 2018).

After having identified these 2 novel *BCL2L12* circRNAs in CRC cell lines using Sanger sequencing of nested PCR products, we followed a holistic approach for the detection of the circular transcripts of this gene. Thus, we implemented long-read sequencing based on nanopore technology to sequence nested PCR amplicons of *BCL2L12* circRNAs. To date, there is a lack of sensitive algorithms able to identify circRNAs in long sequencing reads obtained following a PCR-based approach; thus, we also developed two sensitive algorithms, to analyze our nanopore sequencing data. After having discovered tens of *BCL2L12* circRNAs in the 7 CRC cell lines and predicted their interactions with particular miRNAs, we sought to answer the question whether a remarkable change in the levels of *BCL2L12* circRNAs comprising part of the 3′-untranslated region (3′-UTR) of *BCL2L12* mRNA could significantly affect the levels of this latter.

## Methodology

### Cell culture

Seven CRC cell lines, namely Caco-2, COLO 205, DLD-1, RKO, HT-29, HCT 116, and SW 620, were propagated according to the ATCC^®^ guidelines, in a cell culture incubator providing a humified atmosphere with 5% CO_2_.

### RNA isolation and cDNA synthesis

Total RNA isolation from the 7 CRC cell lines was performed after cell lysis in TRI Reagent^®^ (Molecular Research Center Inc., Cincinnati, OH, USA), following the manufacturer’s instructions. A spectrophotometer was used to measure the concentration and purity of the extracts. Next, the extracts were loaded on an agarose gel, to assess their integrity. Then, 2 µg of each RNA extract were used as a template for cDNA synthesis. First-strand cDNA was synthesized using M-MLV Reverse Transcriptase (Invitrogen™, Thermo Fisher Scientific Inc., Carlsbad, CA, USA) and 50 ng of random hexamer primers (New England Biolabs Ltd., Hitchin, UK) in a final reaction volume of 20 µL, following the standard protocol.

### Divergent primer designing, PCR assays and product purification

Each cDNA was used as a template to perform a first-round PCR assay with divergent primers, specifically designed for each *BCL2L12* exon (Fig. S1A); their sequences are given in Table S1. The first-round PCR products were diluted 50-fold and used as a template in a second-round (nested or semi-nested) PCR assay, again using divergent primers (Table S1). Equal masses of the second-round PCR products of each cell line were mixed and purified with the use of spin columns (Macherey-Nagel GmbH & Co. KG, Düren, Germany). The concentration of the unpurified and mixed purified PCR products was determined using a Qubit 2.0 fluorometer (Invitrogen™). More details about the reaction mixtures and the thermal protocols can be found in Supplementary materials and methods.

### Nanopore sequencing

DNA libraries were prepared according to the standard protocol provided by Oxford Nanopore Technologies plc (Oxford, UK), using 70 fmol of each purified PCR product mix. In brief, the PCR products were end-repaired and barcoded by ligation. Next, sequencing adapters were ligated to each barcoded mix of amplicons, thus providing the nanopore sequencing library, which was then sequenced in a suitable flow cell with the Flongle adapter adjusted on a MinION Mk1C platform (Oxford Nanopore Technologies plc). More details about reagents used for nanopore sequencing can be found in Supplementary materials and methods.

During the sequencing run, 34,180 reads were generated, covering a total of 15.67 megabases (Mb). The number of total reads plotted against sequencing run time as well as the number of reads generated for each cell line are shown in Fig. S2.

### Nanopore sequencing data analysis

All reads were mapped using Minimap2 (Li 2018), against chromosome 19 (chr19) of the *Homo sapiens* genome assembly GRCh38.p14 (hg38), to avoid erroneous alignment to *BCL2L12P1* processed pseudogene sequence. The produced SAM files were next used to generate BAM files and their indexes utilizing samtools, and to also measure the coverage of *BCL2L12* genomic region by sequencing reads (Danecek et al. 2021). The SAM files were also used with samtools to produce new FASTQ files, containing only the aligned reads; these FASTQ files were those used further, as described below.

As a next step, we used the ASDT algorithm (version 2.1) for sensitive detection of splicing events (Adamopoulos et al. 2018). However, this algorithm could not detect back-splice junctions; for this reason, we used multiple modified GenBank^®^ record (“.gb”) files, each one deriving from *BCL2L12* gene record of GenBank^®^ by rearranging the exon order in the gene sequence so that the reconstructed sequence would comprise the same exon at both the beginning and the end of *BCL2L12* genomic sequence. The ASDT algorithm was applied to the new FASTQ files along with each modified “.gb” file by using strict criteria; specifically, each splice junction was identified only if 20 nucleotides in a row around the junction were detected, without any mismatch being allowed.

Next, a new Perl script (ASDT remodeler) was compiled, to recognize and discern the reads representing circRNAs. Moreover, the previously created BAM files were visually inspected using the Integrative Genomics Viewer (IGV) (Robinson et al. 2011) to find reads representing single-exon circRNAs. Manual annotation of indicative reads of single-exon and multi-exon circRNAs was then performed. All novel circRNA sequences were collected in a FASTA file, which was aligned with minimap2 against chromosome 19 (chr19) of the *Homo sapiens* genome assembly GRCh38.p14 (hg38); the produced SAM file was converted to a BAM file along with its index file, using samtools, and finally a BED file was generated, using BEDtools (Quinlan and Hall 2010).

Lastly, we aimed to investigate the expression profile of the novel *BCL2L12* circRNAs among the 7 CRC cell lines, based on our nanopore sequencing data. For this reason, another PERL script (Read catcher) was developed; this algorithm searches for specific keywords in a FASTQ file. Therefore, we used the sequence of each BSJ as a keyword to search for each novel circRNA in the sequencing dataset of each CRC cell line. A cumulative diagram illustrating our bioinformatics pipeline is shown in Fig. S1B.

### BSJ confirmation by next-generation sequencing (NGS)

Specific divergent primers spanning each novel BSJ were designed, with the sole exception of the BSJ involving a poly(A) tract, present only in circ-BCL2L12-9 and circ-BCL2L12-55. A 25-cycle pre-amplification and a semi-nested real-time PCR with SYBR^®^ Green chemistry were conducted using these BSJ-specific and other primers shown in Table S2. Each BSJ was detected in a pool of cDNAs from the CRC cell lines in which it had initially been detected using nanopore sequencing. The specificity of the amplicons was assessed through agarose gel electrophoresis, based on their length. Moreover, to confirm the BSJ of the circRNAs comprising a poly(A) tract, a specific amplicon spanning the respective BSJ was produced via nested PCR, electrophoresed, gel-extracted, and purified using a spin column (Macherey-Nagel GmbH & Co. KG), and then subjected to Sanger sequencing.

To further validate the identified BSJs, 2 µg of total RNA extracted from each CRC cell line was treated with RNase R (Abcam Inc., Cambridge, UK) prior to reverse transcription using the highly thermostable Maxima™ H Minus Reverse Transcriptase (Thermo Scientific™, Thermo Fisher Scientific Inc., Waltham, MA, USA), in order to avoid template switching in reverse transcription. The effectiveness of RNase R treatment was validated by a significant reduction in *GAPDH* mRNA levels, quantified using a standard real-time quantitative PCR (qPCR) protocol after reverse transcription following RNase R treatment (Kontos et al. 2008), compared to a cDNA produced by reverse transcription not preceded by RNase R treatment (Fig. S3A). On the other hand, as might be expected, no significant reduction was observed in ciRS-7 after RNase R treatment (Fig. S3B). Next, a 25-cycle pre-amplification and a semi-nested real-time PCR with SYBR^®^ Green chemistry were conducted using these BSJ-specific and other primers shown in Table S2. Again, each BSJ was detected in a pool of cDNAs from the CRC cell lines in which it had initially been detected using nanopore sequencing. Moreover, to further validate the BSJ of the circRNAs comprising a poly(A) tract, a specific amplicon spanning the respective BSJ was produced via nested PCR. The specificity of all amplicons was primarily assessed through agarose gel electrophoresis, based on their length. Next, equal masses of all BSJ-specific amplicons as well as the one comprising the poly(A) tract were pooled together and subjected to next-generation sequencing (NGS) in a MiSeq System (Illumina, San Diego, CA, USA), using 150-bp paired-end (PE150) sequencing chemistry. The resulting FASTQ file was aligned against the expected sequences of the amplicons of interest, using the STAR aligner (Dobin et al. 2013).

Lastly, as part of the validation process, aligned reads (BAM files) were obtained from the ENCODE database, specifically generated using the same NGS datasets that had been employed for the construction of circBase (Glazar et al. 2014). These BAM files were generated by aligning the reads against *Homo sapiens* genome assembly GRCh37 (hg19) and used for the extraction of reads specifically aligning to the *BCL2L12* gene, using samtools (Danecek et al. 2021). The new BAM files containing these NGS reads were used to produce respective FASTQ files, on which the Read_catcher algorithm was applied to detect and hence verify the novel BSJs. Finally, manual annotation of representative NGS reads was performed, to ensure the accuracy of the BSJ sequence detection.

More details about all reaction mixtures and thermal protocols can be found in Supplementary materials and methods.

### Bioinformatic prediction of miRNA-binding sites in *BCL2L12* circrnas

The novel *BCL2L12* circRNA sequences comprising the annotated 3′-UTR of *BCL2L12* mRNA (or a part of it) were bioinformatically scanned for miRNA-binding site detection, using the miRDB custom prediction tool (Chen and Wang 2020).

### Plasmid construction, bacteria transformation, and plasmid purification

The psiCHECK-2 vector (Promega, Madison, WI, USA) was digested using NotI and XhoI restriction enzymes (New England Biolabs Ltd). In brief, 1 µg of the vector was digested, using 20 units of each of the NotI and XhoI restriction enzymes, in rCutSmart buffer (New England Biolabs Ltd.). The reaction mixture was incubated at 37 ^o^C for 60 min, followed by heat inactivation at 65 ^o^C for 20 min. Next, the reaction mixture was loaded on a 0.8% agarose gel, and the linearized plasmid bands were excised and purified using QIAEX II Gel Extraction Kit (Qiagen GmbH, Hilden, Germany). The purified linearized plasmid was then ligated with a double-stranded DNA sequence comprising the concatenated BSJs (40 bp each) of the 2 circRNAs of interest, namely circ-BCL2L12-29 and circ-BCL2L12-92; a second recombinant plasmid containing the annotated *BCL2L12* 3′-UTR was also constructed. Recombinant plasmid construction was conducted using the NEBuilder^®^ HiFi DNA Assembly Cloning Kit (New England Biolabs Ltd), 0.016 pmol of the linearized vector, and 0.083 pmol of each insert, following the manufacturer’s protocol.

Then, 2 µL of the ligation reaction mixture was used to transform NEB^®^ 5-alpha Competent *E. coli* cells (New England Biolabs Ltd). After transformation, the bacteria mix was spread in Luria Broth (LB) agar selection plates containing carbenicillin and incubated at 37^o^C for 16 h. Next, colonies were inoculated with liquid bacteria cell culture, in 50 mL of LB medium containing carbenicillin, and incubated in a shaking incubator at 37^o^C for 16 h.

Lastly, plasmid purification was performed using ZymoPURE II Plasmid Midiprep Kit (Zymo Research Europe GmbH, Freiburg, Germany).

### Dual-luciferase reporter assay

Two small interfering RNA (siRNA) duplexes, one specific for the BSJ of circ-BCL2L12-29 and another one for circ-BCL2L12-92, were designed (Table S3); a scrambled duplex was also used. To perform a dual-luciferase reporter assay, 9.5 × 10^4^ HCT 116 cells were seeded in each well of a 96-well plate. After 24 h, 50 ng of each plasmid construct were transfected in respective wells, using the jetPRIME^®^ transfection reagent (Polyplus, Illkirch, France). After a 2-hour incubation, 2 pmol of the siRNAs were transfected in each well, using Lipofectamine RNAiMax (Invitrogen™). Besides those wells containing either of the plasmid constructs and one of the siRNAs, wells containing the plasmid construct and the scrambled duplex were included for normalization, as well as wells including only the transfection reagents (mock control). Each reaction was performed in triplicate. Twenty-four (24) hours after siRNA transfection, quantification of the luminescent signal of Renilla and firefly luciferases was conducted. In brief, the Dual-Glo^®^ Luciferase Assay System (Promega) was used for the quantitation of luminescence in a Cytation 5 Cell Imaging Multimode Reader (BioTek, Agilent Technologies, Winooski, VT, USA), following the standard protocol.

### SiRNA transfection, RNA isolation, and first-strand cDNA synthesis

HCT 116 cells were seeded in 12-well plates, at a concentration of 1.2 × 10^5^ cells per well. After 24 h, cells were transfected with 10 pmol of each siRNA duplex, including the scrambled one, using Lipofectamine RNAiMax (Invitrogen™). Wells comprising only transfection reagents (mock control) and wells with untransfected HCT 116 cells for normalization were included as well. Each reaction was performed in triplicate. The cells were incubated with transfection reagents for 24 h. Next, RNA isolation was performed using RLT lysis buffer (Qiagen GmbH) and ZymoSpin columns (Zymo Research Europe GmbH). Lastly, 100 ng of each total RNA extract were used to perform first-strand cDNA synthesis, using iScript cDNA synthesis kit (Bio-Rad Laboratories, Hercules, CA, USA).

### Pre-amplification and real-time qPCR of circ-BCL2L12-92 and *BCL2L12* mRNA

Due to the low expression levels of the *BCL2L12* circRNAs, pre-amplification of circ-BCL2L12-92 and ciRS-7 (reference circRNA) was needed. For this reason, a 20-cycle PCR was conducted, using Taq DNA polymerase and the Thermopol Buffer (New England Biolabs Ltd). The primer pairs and annealing temperatures are shown in Table S4. The PCR products were diluted 50-fold to serve as templates for real-time qPCR. Next, nested real-time qPCR assays were developed for both circRNAs, resulting in the relative quantification of circ-BCL2L12-92. Moreover, real-time qPCR was used to quantify *BCL2L12* mRNA expression, normalized against *GAPDH*, *ACTB* and *ACTG1*, which served as reference genes for mRNA quantification. More details about the reaction mixtures and the thermal protocols can be found in Supplementary materials and methods.

The specificity of the qPCR products was assessed by checking the amplicon size via agarose gel electrophoresis. Real-time qPCR assay development also included the generation of a standard curve for each amplicon, using serial template dilutions as input; each reaction was performed in triplicate to ensure data reproducibility. The expression levels of circ-BCL2L12-92 and *BCL2L12* mRNA were determined using the relative quantification (2^−∆∆Ct^) method (Livak and Schmittgen 2001; Schmittgen and Livak 2008). ciRS-7 served as an endogenous control for circ-BCL2L12-92, whereas *GAPDH*, *ACTB* and *ACTG1* mRNAs served as references for *BCL2L12* mRNA; the cDNA from untransfected HCT 116 cells was used as a calibrator. The mean expression of each of circ-BCL2L12-92 and *BCL2L12* mRNA along with its standard error were calculated using 3 biological replicates.

## Results

### Development of a new experimental and bioinformatic workflow for circrna identification

Our study proposes a new workflow for the identification of novel circRNAs deriving from a single gene (Fig. S4). In brief, after RNA extraction from 7 CRC cell lines and cDNA synthesis, we designed 2 pairs of divergent primers annealing to each *BCL2L12* annotated exon, to perform nested PCRs. The distinct PCR products from each cell line were mixed, purified, and used for nanopore sequencing library construction, using a specific barcode for each cell line. After nanopore sequencing of the amplicon mixture, extensive bioinformatic analysis using publicly available tools and our own Perl-based algorithms was performed. ASDT, a highly sensitive algorithm for the detection of alternative transcripts, was modified and used by applying strict criteria (Adamopoulos et al. 2018). Moreover, as “ASDT” is only able to detect linear transcripts, a couple of additional algorithms, namely “ASDT remodeler” and “Read catcher” were developed to detect and discern reads representing circRNAs. Lastly, the sequences of the novel circRNAs were manually annotated, one by one.

### Exon structure and characteristics of the novel *BCL2L12* circrnas

In our study, 46 *BCL2L12* circRNAs were identified in the CRC cell lines, one of which (circ-BCL2L12-1) had already been identified in a previous study of our research group (Karousi et al. 2020). Approximately half of the *BCL2L12* gene was shown to be cumulatively covered by the identified circRNAs, while its longest mature mRNA covers 21% of the same genomic region. The identified *BCL2L12* circRNAs have been deposited in GenBank^®^ of NCBI.

**Fig. 1:**
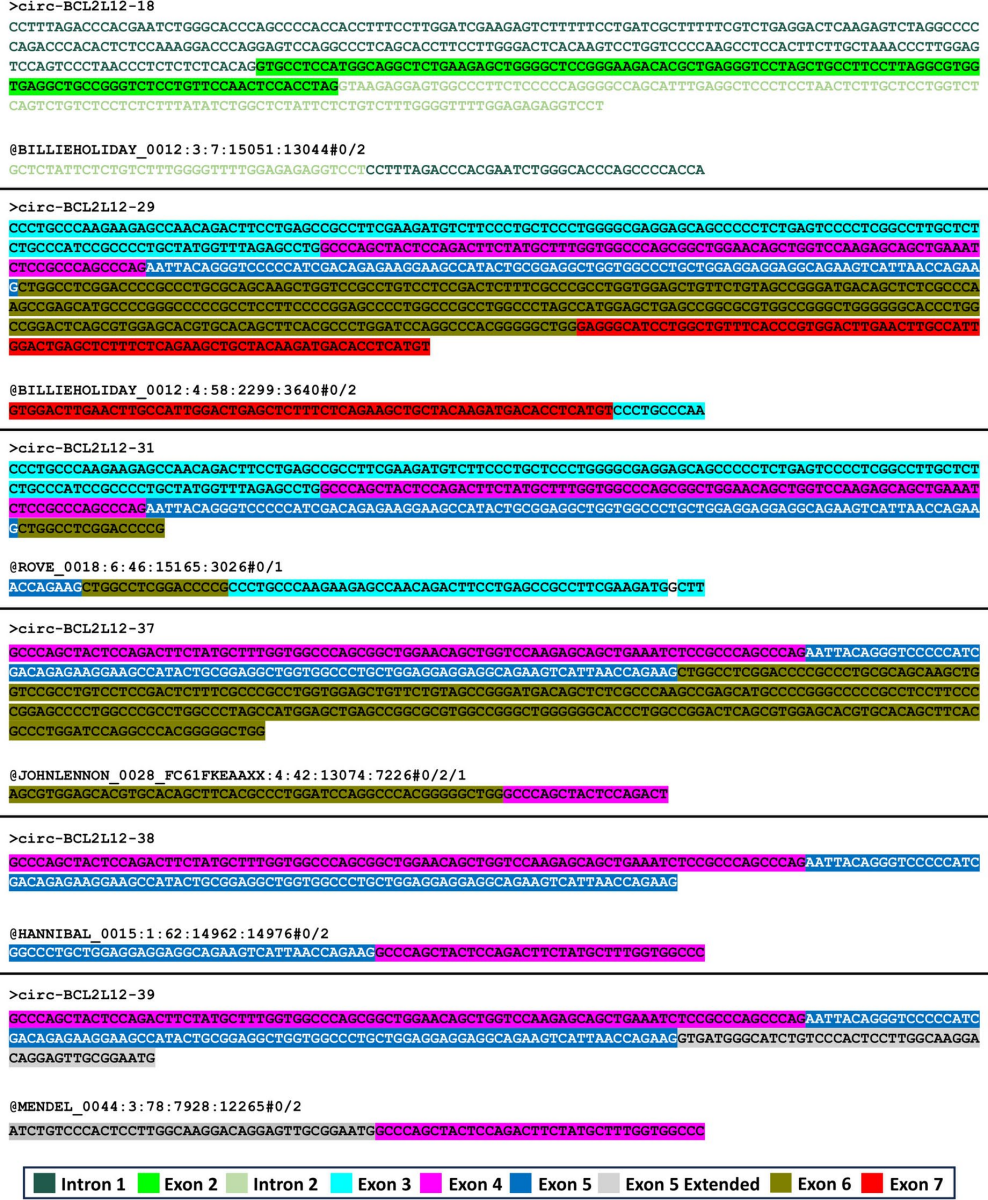
Annotated reads representing the back-splice junctions (BSJs), obtained from bioinformatics analysis of the publicly available RNA-seq datasets previously used to deduce full-length circRNA sequences currently deposited in public circRNA repositories. Non-colored text indicates a mismatch between the read and the reference sequence

To date, only a handful of circRNAs deriving from the *BCL2L12* gene are listed in public repositories, including CircAtlas 3.0, CIRCpedia v.2, and circBase (Dong et al. 2018; Glazar et al. 2014; Wu et al. 2020). Among the 46 *BCL2L12* circRNAs we identified, only 3 were found to be previously deposited in these repositories. Specifically, circ-BCL2L12-37 and circ-BCL2L12-39 have been recorded in CIRCpedia v.2 as HSA_CIRCpedia_87899 and HSA_CIRCpedia_26456, respectively. Additionally, circ-BCL2L12-38 has been documented in circBase as hsa_circ_0051933, in CircAtlas 3.0 as hsa-BCL2L12_0001, and in CIRCpedia v.2 as HSA_CIRCpedia_26455. Although only circ-BCL2L12-38 had been deposited in circBase, our comprehensive bioinformatics analysis of the same datasets employed by circBase revealed the presence of 5 additional novel circRNAs, namely circ-BCL2L12-18, circ-BCL2L12-29, circ-BCL2L12-31, circ-BCL2L12-37, and circ-BCL2L12-39 (Fig. 1).

**Fig. 2:**
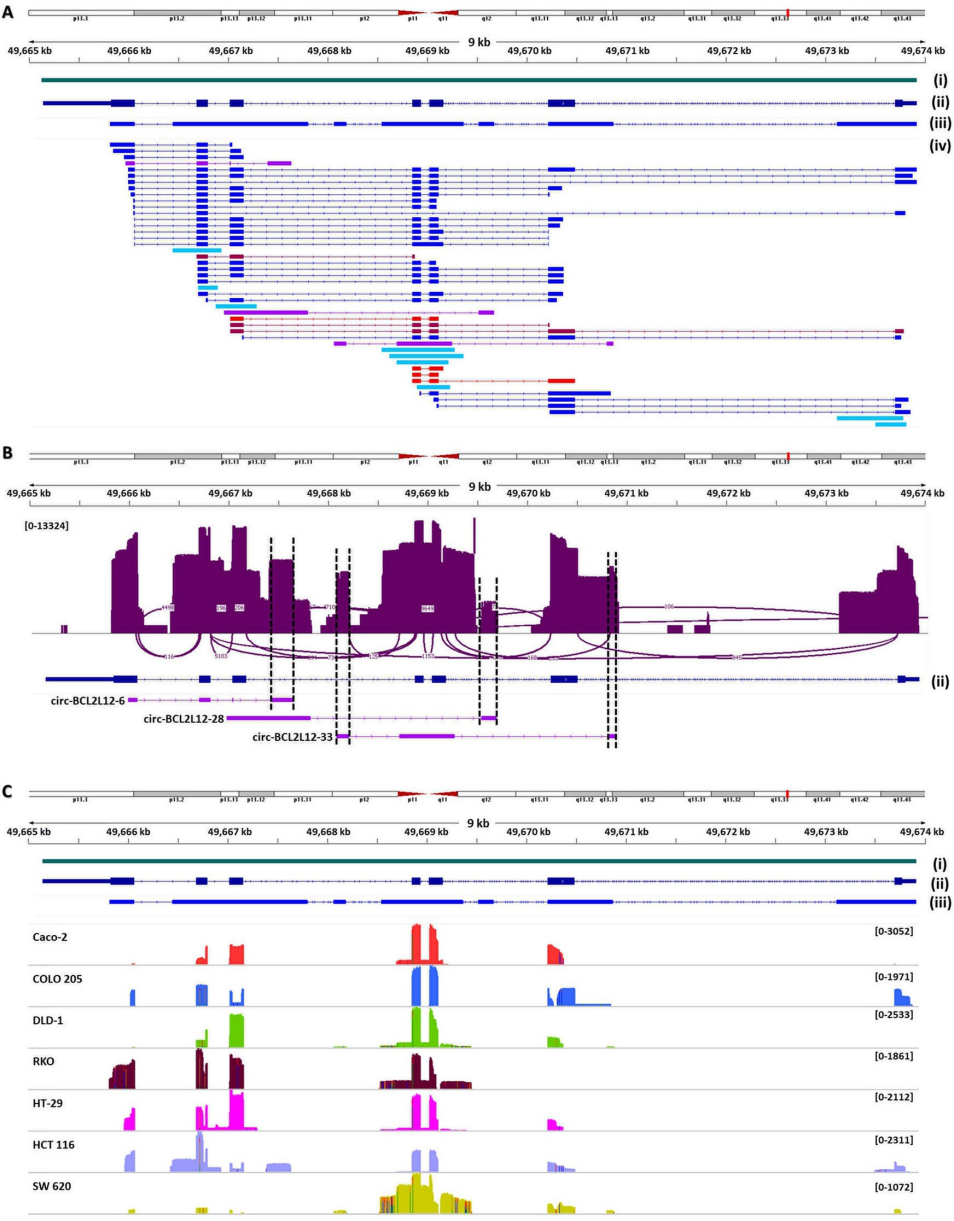
Representation of *BCL2L12* circRNAs and their nanopore sequencing reads in the 7 colorectal cancer (CRC) cell lines, using the Integrative Genomics Viewer (IGV). (**A**) The alignment of *BCL2L12* circRNAs against chromosome 19 (chr19) of the *Homo sapiens* genome assembly GRCh38.p14 (hg38) was done using Minimap2. The circRNAs cumulatively (iii) cover a much wider region of the primary transcript of this gene (i), compared to all mRNAs together (ii). The acceptor back-splice site of each circRNA constitutes the starting position of each aligned sequence (iv). Distinct colors of aligned sequences indicate subgroups; cyan: single-exon circRNAs; purple: circRNAs comprising one or two novel cryptic exons; jazzberry jam and red: circRNAs with one or both (respectively) back-spliced exons being already known and of full length. (**B**) This Sashimi plot demonstrates the plurality of splice junctions formed between usual or cryptic splice sites, including those of 4 novel cryptic exons harbored by *BCL2L12* introns. Only the most frequent splice junctions (covered by > 50 reads) are shown. The vertical axis showing the coverage is drawn in *log* scale. (**C**) The coverage of the *BCL2L12* genomic region by the nanopore sequencing reads of circRNAs differed among the 7 CRC cell lines. Intronic regions of *BCL2L12* are much less represented than all known exons of this gene. The numbers in parentheses indicate the frequency range

Regarding the exon structure of the novel circRNAs, nine of them consisted of a single exon. All of them include at least one annotated exon plus part of the flanking introns, as their initial amplification was based on exon-specific primers. The single-exon *BCL2L12* circRNAs are highly overlapping, e.g. circ-BCL2L12-34, circ-BCL2L12-35a, circ-BCL2L12-36, and circ-BCL2L12-40 (Fig. 2A). Moreover, 3 circRNAs comprised 1 or 2 exons that had never been described before. A characteristic distinguishing these 4 novel exons from other intronic regions exploited in circRNAs is their frequency. Therefore, as clearly illustrated in Fig. 2B, the coverage of these 4 cryptic exons is lower than all previously annotated exons but higher, at the same time, than all other intronic regions.

Moreover, a BSJ between two known exons of this gene were encountered only in 4 circRNAs (circ-BCL2L12-30, circ-BCL2L12-37, circ-BCL2L12-38, and circ-BCL2L12-39). Lastly, in 3 circRNAs (circ-BCL2L12-19, circ-BCL2L12-29, and circ-BCL2L12-31), the “first” aligned exon was an already annotated one (exon 2 or 3) whereas the “last aligned” exon was a 3′-truncated form of a known *BCL2L12* exon (exon 4, 6, or 7).

### Expression profiling of *BCL2L12* circrnas in the CRC cell lines

Nanopore sequencing data analysis also revealed the expression profile of *BCL2L12* circRNAs in the 7 CRC cell lines (Table 1). The coverage of *BCL2L12* genomic region by the circRNAs expressed in each of the 7 CRC cell lines is remarkably different, as clearly illustrated in Fig. 2C. *BCL2L12* exons 4 and 5 are the most encountered among all exons of the gene in circRNAs expressed in all cell lines; however, the exon structure of circRNAs bearing these two exons differ among the 7 CRC cell lines. Apart from that, their intervening intron is ubiquitously present yet much less frequent in HCT 116 and COLO 205 cells. Furthermore, intronic regions are covered in a discernible way among the 7 CRC cell lines. This coverage is usually higher in the flanking intronic regions of already known exons of *BCL2L12*. Only the small introns of this gene– those between exons 2 and 3 and between exons 4 and 5– are fully retained in some circRNA sequences, whilst all introns are partially covered by circRNA-derived sequencing reads.

**Table 1:** Expression analysis of the novel circrnas, discovered by targeted nanopore sequencing, in colorectal cancer (CRC) cell lines

circRNA	GenBank^®^ accession #	Expression^1^						
		Caco-2	COLO 205	DLD-1	RKO	HT-29	HCT 116	SW 620
circ-BCL2L12-1	ON142203.1	–	+	+	+	–	+	–
circ-BCL2L12-2b	ON141952.1	+	–	–	–	–	–	+
circ-BCL2L12-3	ON141935.1	–	–	–	+	–	–	–
circ-BCL2L12-4	ON141936.1	–	–	–	+	–	–	–
circ-BCL2L12-5	ON141937.1	–	–	–	–	+	–	–
circ-BCL2L12-6	ON141938.1	–	–	–	–	–	+	–
circ-BCL2L12-8	ON141940.1	–	+	–	–	–	–	–
circ-BCL2L12-9	ON141941.1	–	+	–	–	–	–	–
circ-BCL2L12-10	ON141942.1	–	+	–	–	–	–	–
circ-BCL2L12-11	ON141943.1	–	–	–	+	+	–	–
circ-BCL2L12-12	ON141944.1	–	–	–	–	–	+	–
circ-BCL2L12-13	ON141945.1	+	–	–	–	–	–	–
circ-BCL2L12-14	ON141946.1	+	–	+	–	–	–	–
circ-BCL2L12-15	ON141947.1	+	–	+	–	–	–	–
circ-BCL2L12-16	ON141948.1	–	–	+	–	–	–	–
circ-BCL2L12-17	ON141949.1	–	–	+	–	–	–	–
circ-BCL2L12-18	ON141950.1	–	–	–	–	–	+	–
circ-BCL2L12-19	ON141951.1	–	–	–	+	–	–	–
circ-BCL2L12-21	ON141953.1	+	–	+	–	–	+	–
circ-BCL2L12-22	ON141954.1	+	–	–	–	–	–	–
circ-BCL2L12-23	ON141955.1	–	–	–	–	–	+	–
circ-BCL2L12-24	ON141956.1	–	–	–	–	+	–	–
circ-BCL2L12-25	ON141957.1	+	–	–	–	–	–	–
circ-BCL2L12-26	ON141958.1	–	–	–	–	–	+	–
circ-BCL2L12-27a	ON141959.1	–	–	–	–	+	–	–
circ-BCL2L12-28	ON141960.1	–	–	–	–	–	+	–
circ-BCL2L12-29	ON141961.1	–	–	–	–	–	+	–
circ-BCL2L12-30	ON141962.1	–	–	–	–	–	+	–
circ-BCL2L12-31	ON141963.1	–	+	–	–	–	+	–
circ-BCL2L12-32	ON141964.1	–	+	–	–	–	–	–
circ-BCL2L12-33	ON141965.1	–	–	+	–	–	–	+
circ-BCL2L12-34	ON141966.1	–	+	+	–	+	+	+
circ-BCL2L12-35a	ON141967.1	–	–	–	+	+	–	–
circ-BCL2L12-36	ON141968.1	+	–	–	–	–	+	+
circ-BCL2L12-37	ON141969.1	–	–	–	–	–	–	+
circ-BCL2L12-38	ON141970.1	–	–	+	–	–	–	–
circ-BCL2L12-39	ON141971.1	–	–	+	–	–	–	–
circ-BCL2L12-40	ON141972.1	–	–	–	–	+	–	–
circ-BCL2L12-41	ON141973.1	–	+	–	–	–	–	–
circ-BCL2L12-42	ON141974.1	–	–	–	–	–	+	–
circ-BCL2L12-43	ON141975.1	–	–	–	–	–	–	+
circ-BCL2L12-44	ON141976.1	–	+	–	–	–	–	–
circ-BCL2L12-46	ON141978.1	+	–	–	–	–	–	–
circ-BCL2L12-47b	ON141979.1	–	–	–	–	–	–	+
circ-BCL2L12-55	OQ317928.1	–	–	–	–	+	–	–
circ-BCL2L12-92	ON746136.1	–	–	–	–	–	+	–

^1^”+” denotes presence and “–“ denotes absence of the respective *BCL2L12* circRNA

### Features of the BSJs of the novel *BCL2L12* circrnas and their relative abundance in the CRC cell lines

In the 46 identified circRNAs, 40 different BSJs were observed since some had the same BSJ but differences in the rest of their exon structure. One of these BSJs was common between the 2 circRNAs comprising a poly(A) tract (circ-BCL2L12-9 and circ-BCL2L12-55) but having a different combination of exons, as explained later in this section.

**Fig. 3:**
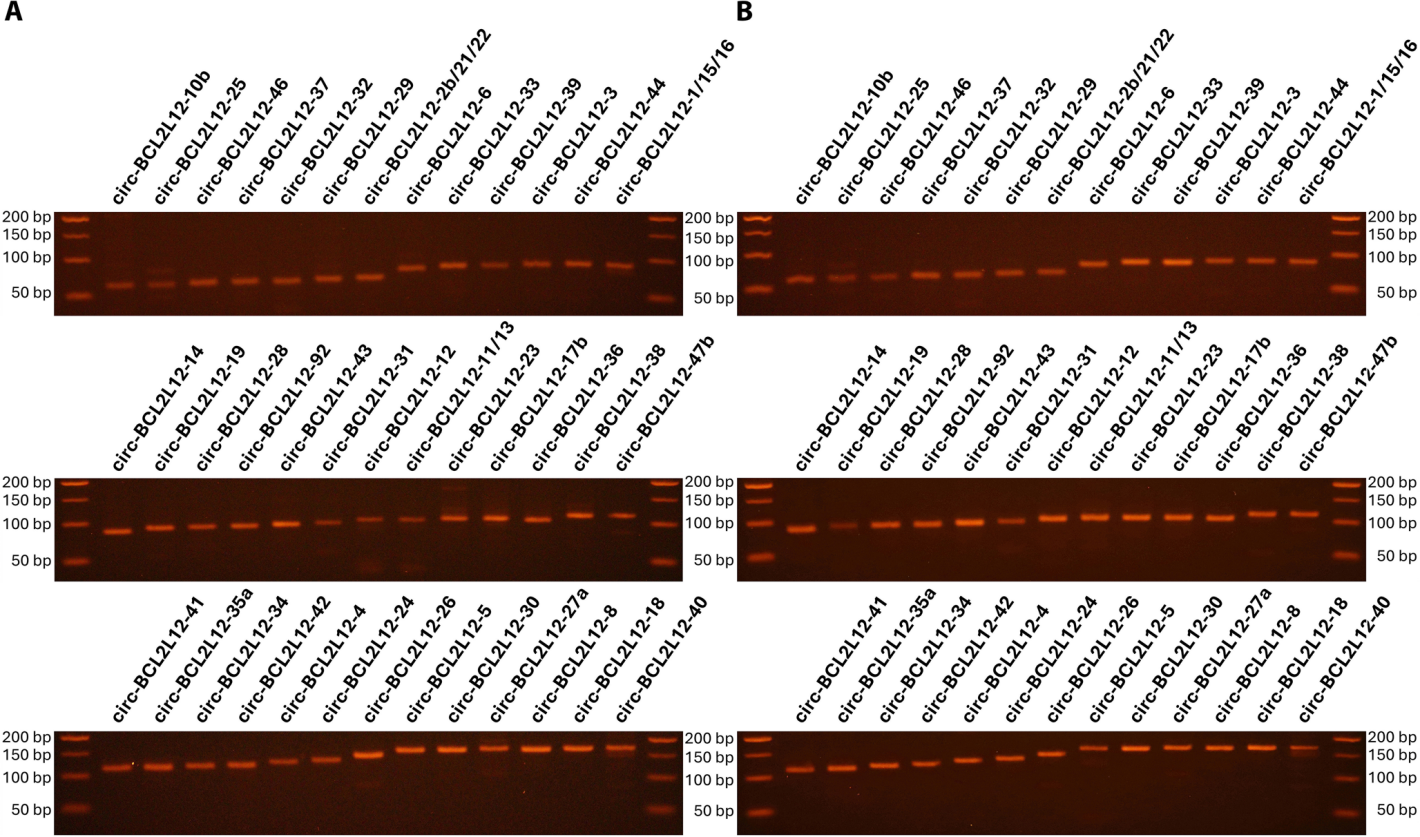
Agarose gel electrophoresis of the back-splice junction (BSJ)-specific amplicons, unique for the vast majority of the identified circRNAs. The cDNA pools used as PCR templates were produced from total RNA extracts of CRC cell lines, without (**A**) or with (**B**) prior RNase R treatment

Additionally, circ-BCL2L12-1, circ-BCL2L12-15, and circ-BCL2L12-16 share the same BSJ; similarly, circ-BCL2L12-2b, circ-BCL2L12-21, and circ-BCL2L12-22 have another common BSJ; lastly, BSJs of circ-BCL2L12-11 and circ-BCL2L12-13 were identical, too. On the other hand, each of the rest 36 *BCL2L12* circRNAs identified in our study was shown to possess a unique BSJ. All these BSJs were primarily confirmed based on their length, as demonstrated via agarose gel electrophoresis of the respective BSJ-specific amplicons produced by semi-nested real-time PCR, starting from pooled cDNAs synthesized from total RNA extracts of CRC cell lines, without or with prior RNase R treatment (Fig. 3A and B, respectively).

In the vast majority of *BCL2L12* circRNAs, the back-splice sites are non-canonical. In fact, besides those 4 circRNAs with 2 known exons being back-spliced together, only 1 more (circ-BCL2L12-36) had canonical (GU– AG) back-splice sites forming the BSJ (Table 2). The non-canonical back-splice sites reside either inside known exons or in introns of primary *BCL2L12* transcripts; thus, their exploitation in the formation of the BSJ leads to extended or truncated exons– sometimes even to microexons– being back-spliced together. On the other hand, the “forward-splice” junctions of the multi-exon *BCL2L12* circRNAs are also encountered in *BCL2L12* mRNAs (Fig. 2A), except for those involving the 4 novel cryptic exons. Given the heterogeneity of forward- and back-splice sites, the plurality of *BCL2L12* circRNAs is not surprising.

**Table 2:** Characteristics of the backs-splice junctions (BSJs) of the novel *BCL2L12* circrnas

circRNA	Genomic coordinates^1^	Back-splice donor site	Back-splice acceptor site	Similarity of pre-mRNA regions^2^ around the back-splice sites
circ-BCL2L12-1, circ-BCL2L12-15, and circ-BCL2L12-16	chr19:49,666,059–49,670,223	CG	CC	GGCCYCGGACC
circ-BCL2L12-2b, circ-BCL2L12-21, and circ-BCL2L12-22	chr19:49,666,695–49,670,375	GC	UG	UGGC
circ-BCL2L12-3	chr19:49,665,813–49,667,047	UU	AC	YAACAGACYY
circ-BCL2L12-4	chr19:49,665,848–49,667,134	AU	CC	
circ-BCL2L12-5	chr19:49,665,958–49,667,158	CC	GA	RRCCYGGY
circ-BCL2L12-6	chr19:49,665,971–49,667,639	UA	GC	AGUGC
circ-BCL2L12-8	chr19:49,665,997–49,673,877	AA	UA	AAUAAA
circ-BCL2L12-9 and circ-BCL2L12-55	chr19:49,665,997–49,673,917	AA	UA	
circ-BCL2L12-10b	chr19:49,666,022–49,670,233	CC	GA	
circ-BCL2L12-11 and circ-BCL2L12-13	chr19:49,666,047–49,669,095	GC	CG	UGGAGGAGG
circ-BCL2L12-12	chr19:49,666,050–49,673,803	GU	CG	
circ-BCL2L12-14	chr19:49,666,059–49,670,335	CG	CC	GCCCCGGRCC
circ-BCL2L12-17b	chr19:49,666,059–49,670,369	CG	CC	CCCG
circ-BCL2L12-18	chr19:49,666,445–49,666,935	CA	CU	UCCUC
circ-BCL2L12-19	chr19:49,666,684–49,668,879	GG	AG	
circ-BCL2L12-23	chr19:49,666,695–49,669,091	GG	UG	
circ-BCL2L12-24	chr19:49,666,702–49,666,899	CU	CU	GGCUCUR
circ-BCL2L12-25	chr19:49,666,702–49,670,365	GG	CU	
circ-BCL2L12-26	chr19:49,666,776–49,670,304	AU	GG	GCCGGG
circ-BCL2L12-27a	chr19:49,666,878–49,667,289	UC	UC	UCCUCUCYYY
circ-BCL2L12-28	chr19:49,666,959–49,669,676	AC	GG	
circ-BCL2L12-29	chr19:49,667,021–49,673,788	CC	AG	CCCUGCCC
circ-BCL2L12-30	chr19:49,667,021–49,669,116	GU	AG	CCAGAAG
circ-BCL2L12-31	chr19:49,667,021–49,670,232	CC	AG	GCCCUGC
circ-BCL2L12-32	chr19:49,667,141–49,673,764	CU	CC	CUGCUAYRR
circ-BCL2L12-33	chr19:49,668,064–49,670,874	CC	AG	AGCCA
circ-BCL2L12-34	chr19:49,668,545–49,669,278	GU	CU	GCUCACGCCUGUAAUCCYAG CACUUUGGGAGGCYGAG
circ-BCL2L12-35a	chr19:49,668,620–49,669,367	CU	AU	CAACAYGGUGAAACCCCAUY UCUACUAAAA
circ-BCL2L12-36	chr19:49,668,697–49,669,215	GU	AG	CAGG
circ-BCL2L12-37	chr19:49,668,850–49,670,489	GU	AG	CYRGGY
circ-BCL2L12-38	chr19:49,668,850–49,669,116	GU	AG	AGGY
circ-BCL2L12-39	chr19:49,668,850–49,669,165	GU	AG	
circ-BCL2L12-40	chr19:49,668,898–49,669,233	AA	AC	GGCUGGRACAR
circ-BCL2L12-41	chr19:49,668,922–49,670,849	CU	AU	AUCUCYRC
circ-BCL2L12-42	chr19:49,669,063–49,673,838	GU	CU	RCUGYGGRG
circ-BCL2L12-43	chr19:49,669,098–49,673,761	AA	AG	CAGAAGYYRYUA
circ-BCL2L12-44	chr19:49,670,232–49,673,854	CC	GC	YYGCCCURC
circ-BCL2L12-46	chr19:49,673,118–49,673,785	UG	CG	CACCUCR
circ-BCL2L12-47b	chr19:49,666,003–49,670,358	AG	UU	
circ-BCL2L12-92	chr19:49,673,501 − 49,673,813	AA	CC	RCYUUUCC

^1^*BCL2L12* circRNA sequences aligned against chromosome 19 (chr19) of the *Homo sapiens* genome assembly GRCh38.p14 (hg38); ^2^Sequences less than 4 nucleotides are not presented; “Y” denotes a pyrimidine and “R” denotes a purine

**Fig. 4:**
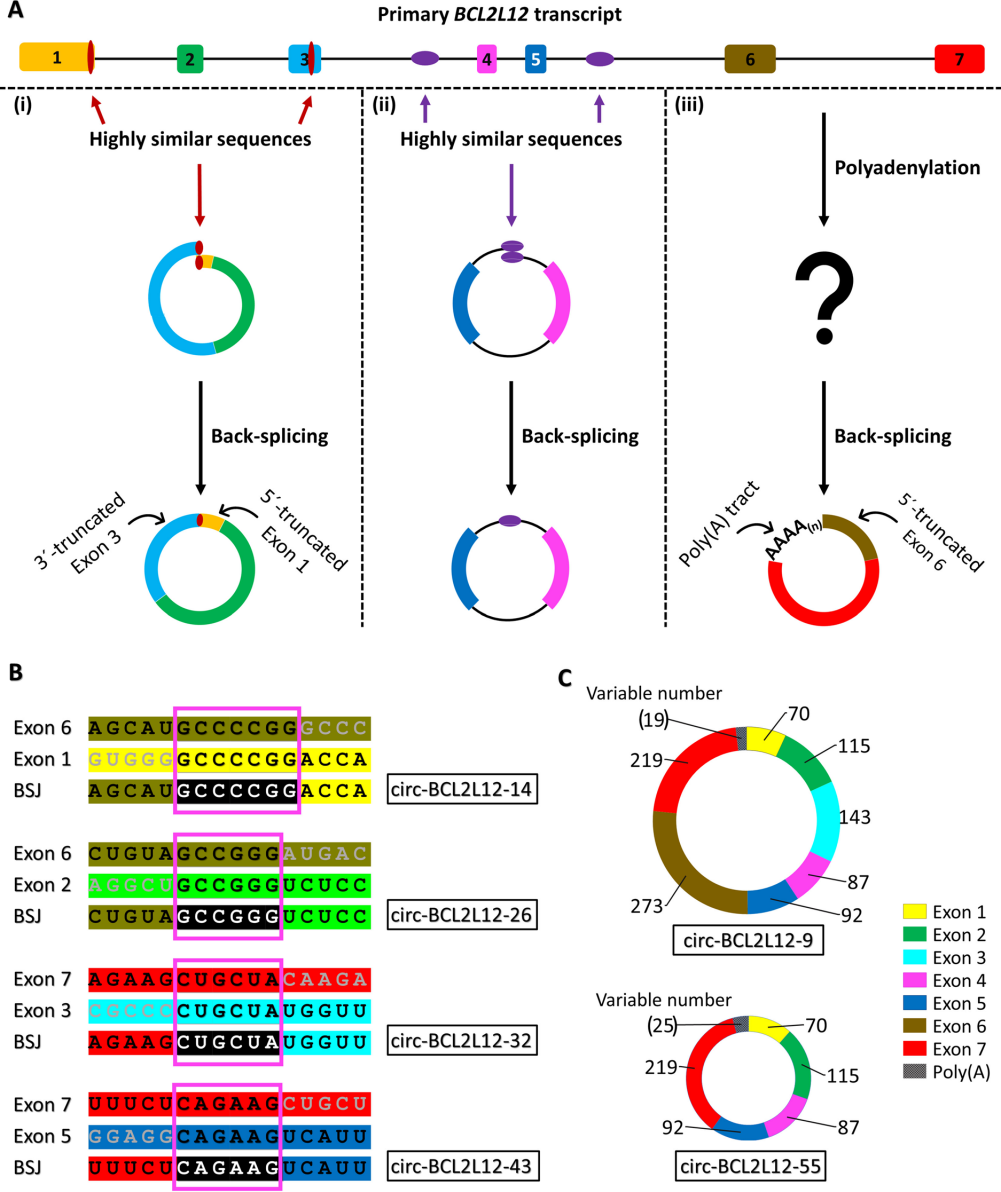
The formation of the back-splice junction (BSJ) between non-canonical back-splice sites in the identified *BCL2L12* circRNAs. **(A)** Two non-canonical back-splice sites residing in highly similar or even identical regions of a primary transcript, either in exons **(i)** or introns **(ii)**, are joined together to form the BSJ of the circRNA. However, a different biogenesis pathway may lead to the formation of circRNA(s) with poly(A) tracts resulting from the poly(A) tail added during the maturation of the primary transcript **(iii)**. **(B)** Only one of the two short identical sequences is present in the circRNA sequence and comprises the BSJ, while the other one is spliced out. **(C)** The *BCL2L12* circRNAs with poly(A) tracts are composed exclusively of the main known exons of this gene (all or most of them)

An intriguing finding was that in most cases (30 out of 40 BSJs), the pre-mRNA regions harboring the non-canonical back-splice sites joined together shared very high local (usually 4–12 nucleotides) sequence similarity (Table 2). Particularly, in circ-BCL2L12-34 and circ-BCL2L12-35a, these highly similar regions in the *BCL2L12* pre-mRNA were 30- and 37-nt long. These highly similar (or even identical) sequences both reside either in the already annotated exons or– in the case of single-exon circRNAs– in introns (Fig. 4A). Furthermore, it was observed that only one of these two regions can be found in the circRNA sequence, while the other one is spliced out (Fig. 4B).

### Inclusion of poly(A) tracts, reminiscent of poly(A) Tails of mRNAs, in two *BCL2L12* circrnas

**Fig. 5:**
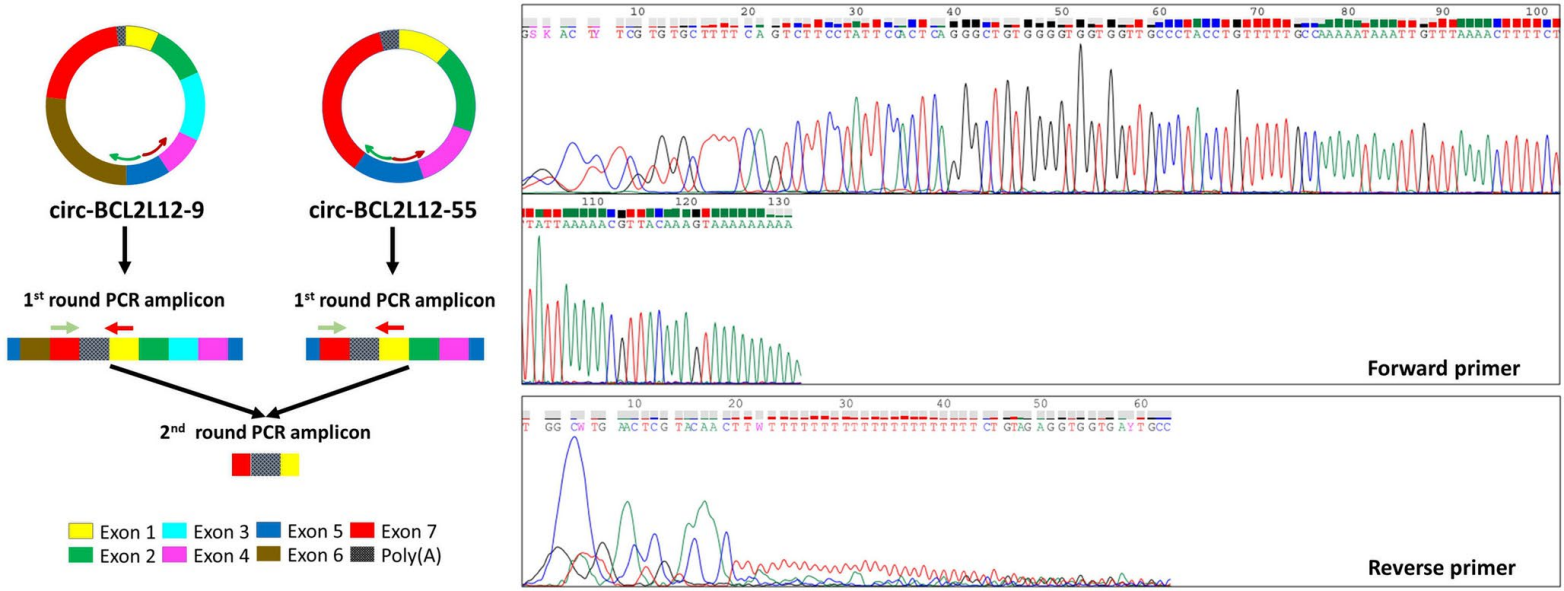
Sanger sequencing chromatograms of a common region of circ-BCL2L12-9 and circ-BCL2L12-55, including the poly(A) tract and the common back-splice junction (BSJ) of these two circRNAs. The green shade-colored arrows indicate the sense primers, whereas the red shade-colored arrows indicate the antisense primers

To date, circRNAs are considered as circular transcripts lacking a 5′ cap and a poly(A) tail, namely two fundamental characteristics of the linear transcripts resulting from maturation of primary transcripts of RNA polymerase II. Surprisingly, a poly(A) tract following exon 7– the last exon of *BCL2L12* mRNAs– was found in 2 novel *BCL2L12* circRNAs (circ-BCL2L12-9 and circ-BCL2L12-55). circ-BCL2L12-9 was found in COLO 205 cells, while circ-BCL2L12-55 was found in the HT-29 cell line. These poly(A) tracts were shown to participate in the BSJ of each of these two circRNAs (Fig. 4A); this finding was also confirmed by Sanger sequencing of a nested PCR amplicon spanning the poly(A) tract and the BSJ (Fig. 5). Of note, both these circRNAs comprise different combinations of known, full-length exons (Fig. 4C).

### Silencing of circ-BCL2L12-92, an exonic-intronic circrna bearing part of *BCL2L12* 3′-UTR, results in downregulation of *BCL2L12* mRNA levels

To explore the putative regulatory role of *BCL2L12* circRNAs regarding the mRNA expression of the parental gene, we sought to downregulate the expression of some of the newly identified molecules including part of the 3′-UTR of the *BCL2L12* mRNA. For this purpose, we decided to use the HCT 116 cell line, in which *BCL2L12* exon 7– comprising the aforementioned 3′-UTR– is widely covered according to our analyzed nanopore sequencing data (Fig. 2C). Four such circRNAs had been identified in this cell line: circ-BCL2L12-12, circ-BCL2L12-29, circ-BCL2L12-42, and circ-BCL2L12-92. After having confirmed with the miRDB tool that all the aforementioned circRNAs share miRNA-binding sites with the annotated *BCL2L12* 3′-UTR using (Table 3), we designed siRNAs against circ-BCL2L12-29 and circ-BCL2L12-92. We focused on these 2 circRNAs since they were more abundant in this cell line, compared to the other 3 aforementioned molecules. A dual-luciferase reporter assay was used to ensure that each of the 2 designed siRNAs could efficiently bind to the BSJ of the targeted circRNAs yet not on the *BCL2L12* 3′-UTR (Fig. S5). This preliminary check led to the exclusion of circ-BCL2L12-29 from further experiments since the siRNA targeting this circRNA was also shown to have an off-target effect on *BCL2L12* mRNA.

**Table 3:** MicroRNAs (miRNAs) predicted to bind both the annotated 3′-untranslated region (3′-UTR) of *BCL2L12* mRNA and some of the novel *BCL2L12* circrnas, detected in the HCT 116 cell line

microRNA	Binding motif	Binding score^1^				
		*BCL2L12* mRNA	circ-BCL2L12-12	circ-BCL2L12-29	circ-BCL2L12-42	circ-BCL2L12-92
miR-455-3p	UGGACUGA	90	76	72	72	75
miR-4264	GACUGAG	85	55	51	52	54
miR-646	AGCUGCUA	78	77	75	74	–
miR-4251	UUCUCAGA	75	75	72	72	74
miR-7-5p	GUCUUCC	72	–	–	69	–
miR-4763-3p	CCCUGCC	64	55	–	56	68
miR-1207-5p	CCCUGCC	64	60	–	56	68
miR-195-5p	GCUGCUA	57	56	53	53	55
miR-6779-3p	CAGGGCU	50	–	–	–	62

^1^Calculated using the miRDB prediction tool (range: 50–100)

**Fig. 6:**
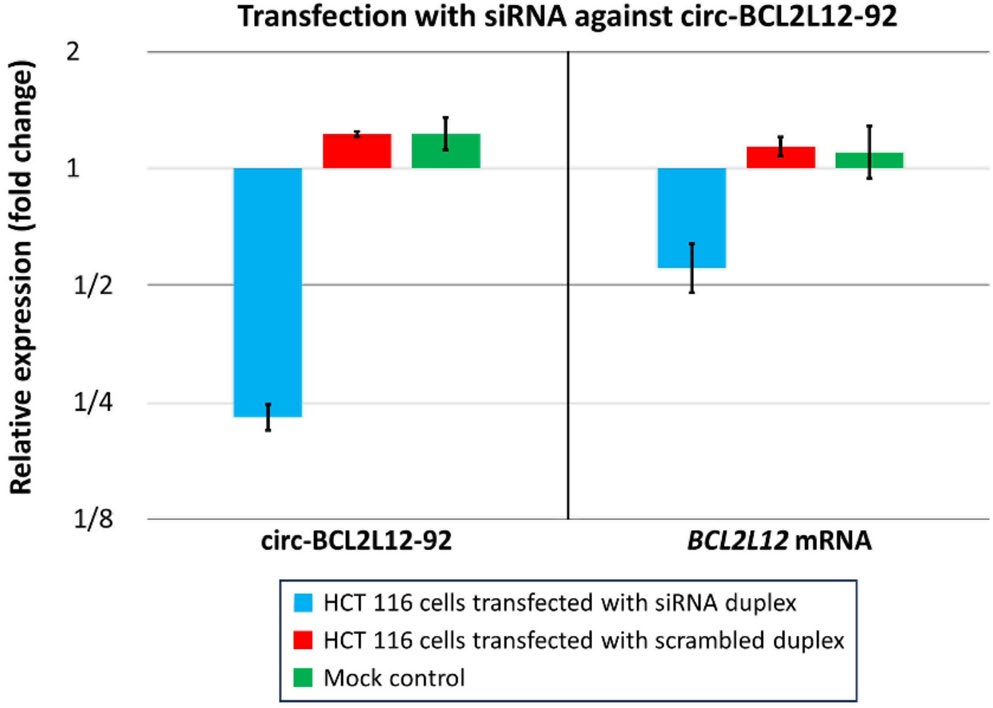
Normalized expression of circ-BCL2L12-92 and *BCL2L12* mRNA in HCT 116 cells transfected with a siRNA duplex targeting the back-splice junction (BSJ) of circ-BCL2L12-92, along with the appropriate controls (HCT 116 cells transfected with scrambled siRNA and untransfected cells). The binding specificity of siRNA against circ-BCL2L12-92 BSJ yet not the 3′-untranslated region (3′-UTR) of *BCL2L12* mRNA was previously demonstrated in a separate experiment. The expression levels of circ-BCL2L12-92 and *BCL2L12* mRNA were determined using the relative quantification (2^−∆∆Ct^) method: ciRS-7 served as an endogenous control for circ-BCL2L12-92, whereas *GAPDH*, *ACTB* and *ACTG1* mRNAs served as references for *BCL2L12* mRNA; the cDNA from untransfected HCT 116 cells was used as a calibrator. The graph shows the mean expression of each of circ-BCL2L12-92 and *BCL2L12* mRNA along with its standard error; 3 biological replicates were used in each case

In brief, after siRNA transfection in HCT 116 cells (3 biological replicates), RNA extraction, cDNA synthesis, pre-amplification of the molecules of interest, and real-time qPCR (Fig. S6), the expression of both circ-BCL2L12-92 and *BCL2L12* mRNA was determined using the relative quantification (2^−∆∆Ct^) method; all reactions were performed in triplicate. The normalization was performed using the appropriate reference genes and untransfected HCT 116 cells as calibrator. Thus, a 75% reduction of circ-BCL2L12-92 levels in HCT 116 cells by its silencing was shown to exert a negative regulatory effect on *BCL2L12* mRNA expression levels, resulting in a ~ 50% decrease (Fig. 6).

## Discussion

Although the splicing process has been studied for years, the full extent of the transcriptome diversity is still questioned, while further investigation still reveals surprising results. Therefore, the advances in high-throughput sequencing techniques provide powerful tools in transcriptomics. NGS technology revolutionized transcriptomics and provided incredible capabilities regarding depth and accuracy; however, this method has some drawbacks regarding the identification of circRNAs, with the most significant one being the short length of obtained reads (Miyamoto et al. 2014). Short-read sequencing approaches can identify BSJs but do not allow the determination of full-length circRNA sequences when such molecules are longer than 500–600 nt (Zheng et al. 2019); these latter can only be deduced by the assembly of short reads. Hence, long-read sequencing is currently the most suitable sequencing method for the identification of full-length circRNAs (Wang et al. 2014).

Although many circRNAs have been identified using massive parallel sequencing, studies have not focused on the transcriptional diversity of a single gene, regarding its circular transcripts. Within this frame, the usage of divergent primer pairs on every exon of a protein-coding gene has not been– to the best of our knowledge– attempted before, as a single pair of divergent primers is usually used, often spanning the BSJ (Panda and Gorospe 2018). Moreover, in this work, we did not follow a circRNA enrichment approach prior to the reverse transcription step. We opted to avoid this step, mainly because most circRNA enrichment processes lead to loss of some circular transcripts (Shi et al. 2022). We also intended to use nested (or semi-nested) PCR products obtained using divergent primers as a template to construct sequencing libraries, as this would ensure the amplification of cDNAs exclusively derived from circRNAs (not from linear RNAs). Moreover, this approach yielded many circRNAs compared to other methods, due to the targeted sequencing we performed instead of RNA-seq; this allowed for very high depth of coverage of the gene of interest. Additionally, our findings were validated using classical techniques for circRNA identification, showing the robustness of our methodology. Regarding the bioinformatic analysis of the nanopore sequencing data, most of the available tools detect circRNAs with annotated exons or, at least, canonical splice sites at exon boundaries (GU as the donor splice site and AG as the acceptor splice site) (Amarasinghe et al. 2020; Rahimi et al. 2021a, b; Szabo and Salzman 2016). However, as we had previously detected– using Sanger sequencing– *BCL2L12* circRNAs with non-canonical splice sites at their BSJ (Karousi et al. 2020; Papatsirou et al. 2022), we aimed at developing a more sensitive and accurate bioinformatics pipeline for the identification of novel *BCL2L12* circRNAs from long-read sequencing data. ASDT is a highly sensitive algorithm for the detection of alternative splicing events, even with unique representation, in a FASTQ file (Adamopoulos et al. 2018); thus, we considered the development of a novel algorithm starting from ASDT v2.1 output files as an optimal option for the discovery of circular *BCL2L12* transcripts with diverse splicing events. Moreover, our newly developed algorithm, “ASDT remodeler”, can be used for the identification of circRNAs deriving from a wide variety of genes, after appropriate modification of the input files. Lastly, the “Read catcher” algorithm enables the detection of these novel molecules in massive parallel sequencing datasets. The sensitivity of the bioinformatics pipeline developed in this study was demonstrated by its ability to identify circRNAs present in the datasets used by circBase yet not deposited in the database, underscoring its efficacy in uncovering previously unrecognized circRNAs.

Even though circular transcripts were discovered years ago, the field of circRNA biology remains unexplored in various aspects. CircRNAs are considered as transcripts with a covalently closed loop, lacking a 5′ cap and a 3′ poly(A) tail (Cocquerelle et al. 1993; Liu et al. 2017; Lu 2020). However, most circRNA enrichment approaches include a removal step of polyadenylated RNA and hence render the identification of circRNAs with poly(A) tracts rather impossible (Lopez-Jimenez et al. 2018). In this work, for the first time, we noticed poly(A) tracts in circRNA sequences. The length of the detected poly(A) tracts varied from 19 to 40 nucleotides; however, as all sequencing methods fail to identify the full extent homopolymers, these poly(A) tracts may be even longer. This is a finding of particular interest, as it raises several questions. As the *BCL2L12* circRNAs bearing such poly(A) tracts do not include intronic regions, it would be expected that their biosynthesis is explained by one of the already described mechanisms of biogenesis of exonic circRNAs (EcircRNAs). However, the inclusion of poly(A) sequences in circRNAs is not supported by any of these mechanisms. More specifically, the currently known mechanisms of circRNA biogenesis are based on base pairing between intronic sequences and/or on the action of RBPs, which bring closer the donor and acceptor splice sites (Geng et al. 2020; Guo et al. 2014; Li et al. 2018). Additionally, the existence of poly(A) tracts in circRNAs poses the issue of the chronological sequence of the RNA processing steps. Thus, it becomes evident that RNA polyadenylation may sometimes precede circularization via the formation of a BSJ. In general, the events of RNA processing are neither chronologically nor topologically discernible (Hocine et al. 2010). It is also important to note that the *BCL2L12* circRNAs with a poly(A) tract were much less abundant than most other circular transcripts of this gene in CRC cell lines, thus raising questions regarding their role as functional molecules or splicing by-products.

Besides the incorporation of poly(A) tracts in novel *BCL2L12* circRNAs, another finding of this work that provides evidence for unexplored circRNA biosynthesis mechanisms is the formation of the BSJ by non-canonical back-splice sites. As already explained, in most of the novel *BCL2L12* circRNAs, the BSJ was formed between non-canonical back-splice sites of the already annotated exons, present in highly similar or even identical regions of primary *BCL2L12* transcripts, usually ranging from 4 to 12 nucleotides. Interestingly, in two cases, these highly similar regions in the *BCL2L12* pre-mRNA were 30- and 37-nt long. The back-splice sites of such circRNAs cannot be annotated unambiguously; however, no matter where exactly one annotates them, the rule of both canonical splice sites (donor “GU” and acceptor “AG) does not seem to apply for most *BCL212* circRNAs. This is not supported by any already proposed mechanism of circRNA biogenesis (Lasda and Parker 2014; Qu et al. 2015). These highly similar sequences could be recognized by RBPs forming homodimers or having twin RNA-binding domains, thus bringing these sequences comprising the back-splice sites in proximity, necessary to form the BSJ. However, unlike in the cases of circMbl (Ashwal-Fluss et al. 2014) and *QKI* circRNA (Conn et al. 2015), one copy of each two highly similar sequences of *BCL2L12* pre-mRNA is retained in *BCL2L12* circRNAs. Moreover, these putative splicing-factor–binding motifs usually reside inside the already annotated exons, although not exclusively; this is another difference with *muscleblind (MBL* / *MBNL1*) and Quaking (*QKI*) pre-mRNAs, in which the respective motifs are intronic (Ashwal-Fluss et al. 2014; Conn et al. 2015). Of note, cryptic splice sites are abundant in cancer cells; their presence results in novel cryptic exons or variations of the already annotated exons (truncations or extensions)– sometimes in microexons as well (Shirley et al. 2018).

In our study, 4 novel exons of the *BCL2L12* gene were discovered in 3 distinct circRNAs, in total. All these exons reside in regions considered to be intronic, as they have not been observed in any linear transcripts so far (Adamopoulos et al. 2016; Kontos and Scorilas 2012). This finding supports the notion that some cryptic exons may be excluded from mRNAs while being part of circRNA(s). Moreover, in combination with the fact that extended “intronic” regions of *BCL2L12* participate in circRNA sequences not merely intronic (ciRNAs) since they constitute wide 5′- or 3′-extensions of known *BCL2L12* exons, it enhances the hypothesis that intronic sequences are usually active, from a functional perspective (Palazzo and Gregory 2014).

Regarding the role of *BCL2L12* circRNAs in CRC, it is intriguing to experimentally decipher their regulatory or protein-coding dynamic. Currently, little is known regarding circRNAs deriving from *BCL2* family members. Such circRNAs are of special interest, due to the established role of their host genes in apoptosis. As the main function of circRNAs is believed to be miRNA sponging (Panda 2018) and, therefore, the fine-tuning of miRNA-mediated post-transcriptional regulation, expression of those circRNAs that comprise common miRNA-binding sites with the mRNAs produced by the same genes can affect the mRNA and/or protein levels. More specifically, miRNAs that bind to the 3′-UTR of *BCL2L12* mRNA could be sequestered by a *BCL2L12* circRNA that shares the same binding sites, thus acting as a competing endogenous RNA (ceRNA). In this work, we provide evidence that circ-BCL2L12-92 can act in such a way, since its silencing by RNA interference (RNAi) indirectly resulted in the downregulation of *BCL2L12* mRNA, as well. As the intrinsic apoptotic pathway is controlled by BCL2 family members (Thomadaki and Scorilas 2006), disruption of such circRNA/miRNA/mRNA axes could affect apoptosis triggering. This makes the elucidation of such regulatory axes particularly important. It should also be noted that circ-BCL2L12-92 could alternatively be achieved by using the CRISPR-Cas13 system (Abudayyeh et al. 2016; East-Seletsky et al. 2016).

Additionally, circRNAs comprising intronic sequences and located in the nucleus have been reported to interact with U1 small nuclear ribonucleoprotein (U1 snRNP) and promote the transcription of their host genes (Huang et al. 2017; Li et al. 2015; Zhang et al. 2013). This is a function of particular interest as well, concerning apoptosis-related genes and several of their circular transcripts; hence, *BCL2L12* circRNAs containing intronic sequences should be further explored in this context, as this could also affect or be linked to CRC progression. Furthermore, the downregulation of circ-BCL2L12-92, which contains intronic sequences, exhibited a significant impact on the expression of the *BCL2L12* mRNA. This finding underscores the regulatory role of this circular RNA in the transcriptional modulation of its host gene, *BCL2L12*, which is known to be associated with apoptosis-related processes. Such intricate regulatory interactions highlight the potential significance of circ-BCL2L12-92 in the context of CRC progression, suggesting that exploring its specific mechanistic contributions could provide valuable insights into the molecular underpinnings of CRC pathogenesis.

Another thought-provoking observation is that *BCL2L12* circRNAs show great diversity in their expression pattern among the 7 studied CRC cell lines. Previous studies have highlighted the tissue– and developmental-stage–specific expression of circRNAs (Xu et al. 2017). Taking into consideration that the majority of *BCL2L12* circRNAs were detected in a single CRC cell line only, it would be interesting to explore the expression patterns of *BCL2L12* circRNAs in other types of cancer as well, in an attempt to conclude whether some of them are tissue- or even cancer-specific.

Undoubtedly, our study has some inherent limitations. Firstly, since the primers used were designed on the currently annotated *BCL2L12* exons, circRNAs deriving merely from intronic regions were not amplified and hence could not be identified. Moreover, since only CRC cell lines were used in the current study, the question of whether normal colorectal epithelial cells express such a wide variety of circular transcripts remains unanswered. Therefore, it would be interesting to investigate whether *BCL2L12* circRNAs identified in cancer cells are also present in non-cancerous colorectal cell lines. Furthermore, although nanopore sequencing has important advantages, it has some drawbacks as well, with the main one being the high error rate until now (Athanasopoulou et al. 2021). This issue could theoretically produce some bias in the determination of the back-splice sites, especially of the less abundant circRNAs. For this reason, we verified the exact sequence of each BSJ using semi-nested PCRs followed by short-read sequencing, with the common primer in both reactions being BSJ-specific for each circRNA. Regarding the nanopore sequencing data analysis, although the pipeline developed is highly sensitive for the identification of novel circRNAs, it is also time-consuming, as it requires increased hands-on time to annotate their sequence. Additionally, the circRNAs identified through this procedure cannot be detected with non-PCR–based approaches due to their low intracellular levels. For the same reason, direct RNA sequencing of these circRNAs is not an option. Lastly, since divergent primers on each exon were used in the nested PCR assays before sequencing library preparation, most circRNAs could have been amplified in more than one reaction. As a result, the relative quantity of circRNAs could not be inferred via nanopore sequencing.

Concluding, in this work, we developed an experimental assay for the detection of circular transcripts deriving from a single gene, as well as a bioinformatics pipeline for the analysis of the targeted long-read sequencing data. In this way, we discovered numerous circular *BCL2L12* transcripts, the expression of which shows great diversity among 7 CRC cell lines. The exon structure of these circRNAs supports the notion that the biogenesis mechanisms and functions of the circRNAs have not been fully elucidated yet. Moreover, the downregulation of a circRNA sharing miRNA-binding sites with *BCL2L12* mRNA led to concomitant downregulation of *BCL2L12* mRNA levels as well, implying the indirect involvement of this novel circRNA in the post-transcriptional regulation of *BCL2L12* expression. Moving forward, it is interesting to explore the regulatory and/or protein-coding potential of these novel circRNAs, to shed light on their functional utility. The role of circRNAs may be more important than we currently think of, also considering their high stability in cells, extracellular vesicles, and body fluids.

## Electronic supplementary material

Below is the link to the electronic supplementary material.

Supplementary Material 1

## Data Availability

The raw nanopore sequencing reads have been deposited to the Sequence Read Archive (SRA) of NCBI, with BioProject accession number PRJNA904232. A modified GenBank^®^ record (“.gb”) file, the “ASDT v2.1” algorithm, the “ASDT remodeler” algorithm, the “Read catcher” algorithm, and examples of their input and output files can be found on GitHub (https://github.com/pkarousi/ASDT_remodeler; https://github.com/pkarousi/Read_catcher). All other data will be made available on request.
