# Supplementary material for: Discovery of circular transcripts of the human BCL2-like 12 (BCL2L12) apoptosis-related gene, using targeted nanopore sequencing, provides new insights into circular RNA biology
Source: Funct Integr Genomics. 2025 Mar 19;25(1):66. doi: 10.1007/s10142-025-01578-1 (PMC11923030; doi:10.1007/s10142-025-01578-1)
Supplement: Supplementary file 1 — Supplementary Material 1 [file 10142_2025_1578_MOESM1_ESM.docx]

**Supplementary materials and methods**

**Divergent primer designing, PCR assays and product purification**

Each cDNA generated from the previous step was used as a template to perform a first-round PCR assay with divergent primers, using KAPA Taq DNA Polymerase (KAPA Biosystems Inc., Woburn, MA, USA) in a MiniAmp Thermal Cycler (Applied Biosystems^™^, Thermo Fisher Scientific Inc., Waltham, MA, USA); specifically, the reaction mixture was composed of 19.4 μL nuclease-free H_2_O, 1X KAPA Taq Buffer, 200 μM of each dNTP, 400 nM of each primer, 0.5 U KAPA Taq DNA Polymerase, and 0.5 μL of cDNA, in a final reaction volume of 25 μL. The thermal protocol was conducted following the manufacturer’s instructions; the cycling step was carried out for 25 cycles, while elongation and final extension steps were carried out at 72 ºC for 90 s. The annealing temperatures (T_a_) are shown in Table S1. Next, the PCR products generated from the first-round PCR assays were diluted 50-fold in nuclease-free H_2_O and used as templates in the second-round (nested or semi-nested) PCR assay, again using divergent primers (Table S1) and the same reaction and thermal conditions as previously.

**Nanopore sequencing**

DNA libraries were prepared according to the protocol provided by Oxford Nanopore Technologies plc (Oxford, UK), using 70 fmol of each previously purified PCR product mix. NEBNext FFPE Repair Mix and NEBNext Ultra II End repair/dA-tailing Module (New England Biolabs Ltd., Hitchin, UK) were used to repair the PCR products and prepare their ends for barcode ligation. Next, different barcodes were ligated to the ends of each end-repaired PCR product mix, using Blunt/TA Ligase Master Mix (New England Biolabs Ltd.) and the Native Barcoding Expansion kit (Oxford Nanopore Technologies plc). Next, sequencing adapters were ligated using the NEBNext Quick Ligation Module (New England Biolabs Ltd.), the Native Barcoding Expansion kit, and the ligation sequencing kit (Oxford Nanopore Technologies plc). A flow cell suitable for the Flongle adapter was primed using the flow cell priming kit (Oxford Nanopore Technologies plc) and the DNA library was used to make the sequencing reaction mixture, using the Flongle Sequencing Expansion kit (Oxford Nanopore Technologies plc). Nanopore sequencing was then performed using the MinION Mk1C platform and the Flongle adapter (Oxford Nanopore Technologies plc).

**BSJ confirmation by next-generation sequencing (NGS)**

A 25-cycle pre-amplification followed by a semi-nested real-time PCR assay based on SYBR Green chemistry was conducted, with specific divergent primers spanning each novel BSJ (Table S2). The pre-amplification reaction mixture included 2.5 μL Η_2_Ο, 1X KAPA SYBR FAST qPCR Master Mix (KAPA Biosystems Inc., Woburn, MA, USA), 200 nM of each primer, and 0.5 μL cDNA, in a final reaction volume of 10 μL. The thermal protocol consisted of an initial denaturation step at 95 ^o^C, followed by 25 cycles of a denaturation step at 95 ^o^C for 3 s and a coupled annealing and extension step at 60 ^o^C for 30 s. All products were then diluted 100-fold. Each circRNA was verified in a pool of cDNAs from the cell lines in which it had already been detected using nanopore sequencing. The semi-nested real-time PCR reaction mixture had the same composition, but the template used was the diluted pre-amplified product. Both reactions were conducted in an QuantStudio^™^ 5 Real-Time PCR System (Applied Biosystems^™^). All semi-nested real-time PCR products were electrophoresed in 3% agarose gels, pre-stained with ethidium bromide, next to the 50 bp DNA Ladder (New England Biolabs Ltd.).

To confirm the BSJ sequence of the circRNAs comprising poly(A) tracts, specific amplicons incorporating the sequences of interest were generated through PCR. Specifically, the nested PCR products having derived from amplification with divergent primers annealing to *BCL2L12* exon 5 (Table S1) were diluted 50-fold and used as a template to conduct an additional PCR. The primer sequences used in this reaction were 5′-GCTGCTACAAGATGACACCTCA-3′ and 5′-CGCCTCCTCCACTCAACT-3′; the reaction mixture was composed of 19.4 μL nuclease-free H_2_O, 1x KAPA Taq Buffer, 200 μM of each dNTP, 400 nM of each primer, 0.5 U KAPA Taq DNA Polymerase, and 0.5 μL of diluted template, in a final reaction volume of 10 μL. The thermal protocol was conducted following the manufacturer’s instructions in a MiniAmp Thermal Cycler (Applied Biosystems^™^); the cycling step was carried out for 35 cycles, with the annealing step being carried out at 60 ^o^C, while the elongation and final extension steps were carried out for 1 min. The PCR products were electrophoresed, gel-extracted and purified using spin columns (Macherey-Nagel GmbH & Co. KG, Düren, Germany), and subjected to Sanger sequencing. The sequences of the primers used in Sanger sequencing were those of the last PCR.

To further validate the identified back-splice junctions (BSJs), 2 μg of total RNA extracted from each CRC cell line was incubated at 37 ^o^C along with RNase R (Abcam Inc., Cambridge, UK), followed by a 15-minute heat inactivation step at 70 ^o^C. The reaction mixture included 1X RNase R buffer, 15 U RNase R, and 20 U Ribolock RNase inhibitor (Thermo Scientific^™^, Thermo Fischer Scientific Inc., Waltham, MA, USA) and nuclease-free H_2_O, in a final reaction volume of 15 μL. Next, the treated RNA extracts were subjected to reverse transcription using Maxima^™^ H Minus Reverse Transcriptase (Thermo Scientific^™^). This reaction mixture included 1X RT Buffer, 20 U Ribolock RNase inhibitor, 100 pmol of random hexamer primer (New England Biolabs Ltd.), 500 μM of each dNTP, 100 U Maxima^™^ H Minus Reverse Transcriptase and 11.5 μL of the RNase-R–treated RNA extract, in a final reaction volume of 20 μL. The thermal protocol was applied according to the manufacturer’s guidelines. Again, a 25-cycle pre-amplification followed by a semi-nested real-time PCR assay based on SYBR Green chemistry was conducted, with specific divergent primers spanning each novel BSJ (Table S2), as described above. The aforementioned nested PCR targeting both circRNAs comprising a poly(A) tract was also performed again, based on the same thermal protocols. The cDNA pools that were used as PCR templates included those that had been generated after RNase R treatment and reverse transcription with the thermostable Maxima^™^ H Minus Reverse Transcriptase (Thermo Scientific^™^). All these PCR products were electrophoresed in 3% agarose gels, pre-stained with ethidium bromide, next to the 50 bp DNA Ladder (New England Biolabs Ltd.), before gel extraction and purification. The purified amplicons were then pooled equimolarly, and this pool was used for next-generation sequencing (NGS) DNA library construction, following a standard protocol, suitable for subsequent NGS in a MiSeq System (Illumina, San Diego, CA, USA), using 150-bp paired-end (PE150) sequencing chemistry.

**Pre-amplification and real-time qPCR of circ-BCL2L12-92 and *BCL2L12* mRNA**

The pre-amplification of the circRNAs was conducted using Taq DNA polymerase and the Thermopol Buffer (New England Biolabs Ltd.), in a T100 Thermal Cycler (Bio-Rad Laboratories Inc., Hercules, CA, USA). The reaction mixture was composed of 20.38 μL nuclease-free H_2_O, 1X ThermoPol Reaction Buffer, 200 μM of each dNTP, 200 nM of each primer, 0.625 U Taq DNA polymerase, and 0.5 μL of each previously synthesized cDNA, in a final reaction volume of 25 μL. The primer pairs and their annealing temperatures are shown in Table S4; the thermal protocol was conducted following the manufacturer’s guidelines, with the extension step being carried out for 1 min. The pre-amplified products were diluted 50-fold to serve as templates for real-time qPCR, which was conducted in a qTOWER^3^ G Real-Time Thermal Cycler (Analytik Jena GmbH, Jena, Germany). The reaction mixture included 2.5 μL Η_2_Ο, 1X iTaq Universal SYBR Green Supermix (Bio-Rad Laboratories), 400 nM of each primer (Table S4), and 0.5 μL of diluted template, in a final reaction volume of 10 μL. The thermal protocol included a denaturation step at 95^o^C for 1 min, followed by 40 cycles of denaturation at 95^o^C for 15 s, and primer annealing and extension at 60^o^C for 1 min. A melt curve was generated after the amplification.

**Supplementary Tables**

**Table S1** First- and second-round PCR primer pairs, used to generate the amplicons serving as templates for nanopore sequencing library construction

|  | **Exon** | **Direction** | **Sequence (5**′🡪**3**′**)** | **T_a_**^1^ **(^o^C)** |
| --- | --- | --- | --- | --- |
| **First-round PCR** | Exon 1 | Forward | GGAGACCGCAAGTTGAGTGG | 60 |
|  |  | Reverse | GTCTCCTCCACTGAACTCGT |  |
|  | Exon 2 | Forward | CCGGGTCTCCTGTTCCAA | 61 |
|  |  | Reverse | CTCACCACGCCTAAGGAAGG |  |
|  | Exon 3 | Forward | CCCTCGGCCTTGCTCTCT | 61 |
|  |  | Reverse | CGAAGGCGGCTCAGGAA |  |
|  | Exon 4 | Forward | CGGCTGGAACAGCTGGTC | 61 |
|  |  | Reverse | GGGCCACCAAAGCATAGAAG |  |
|  | Exon 5 | Forward | TGGAGGAGGAGGCAGAAGTC | 60 |
|  |  | Reverse | TCCGCAGTATGGCTTCCTTC |  |
|  | 3′-extended exon 5 | Forward | GCATCTGTCCCACTCCTTGG | 60 |
|  |  | Reverse | TCCGCAGTATGGCTTCCTTC |  |
|  | Exon 6 | Forward | TGTTCTGTAGCCGGGATGAC | 60 |
|  |  | Reverse | GCGAAAGAGTCGGAGGACAG |  |
|  | Exon 7 | Forward | ACTTGCCATTGGACTGAGCTCT | 60 |
|  |  | Reverse | TCAAGTCCACGGGTGAAACA |  |
| **Second-round PCR** | Exon 1 | Forward | AAGTTGAGTGGAGGAGGCG | 61 |
|  |  | Reverse | CCTCCACTGAACTCGTACAAACT |  |
|  | Exon 2 | Forward | CTCCTGTTCCAACTCCACCTAG | 61 |
|  |  | Reverse | AAGGAAGGCAGCTAGGACC |  |
|  | Exon 3 | Forward | TCCGCCCCTGCTATGGTTTA | 61 |
|  |  | Reverse | CTGTTGGCTCTTCTTGGGCA |  |
|  | Exon 4 | Forward | CAGCTGAAATCTCCGCCCAG | 61 |
|  |  | Reverse | GCATAGAAGTCTGGAGTAGCTGG |  |
|  | Exon 5 | Forward | GGAGGCAGAAGTCATTAACCAGAAG | 61 |
|  |  | Reverse | TCCGCAGTATGGCTTCCTTC |  |
|  | 3′-extended exon 5 | Forward | CTTGGCAAGGACAGGAGTTG | 61 |
|  |  | Reverse | TCCGCAGTATGGCTTCCTTC |  |
|  | Exon 6 | Forward | CGCCCAAGCCGAGCAT | 61 |
|  |  | Reverse | GCGAAAGAGTCGGAGGACAG |  |
|  | Exon 7 | Forward | GCTGCTACAAGATGACACCTCA | 61 |
|  |  | Reverse | GAAACAGCCAGGATGCCCTC |  |

^1^Annealing temperature.

**Table S2** Divergent primers used to validate the novel circRNAs, based on their back-splice junction (BSJ)

| **circRNA** | **Primer sequence (5**′🡪**3**′**)** | | | **Amplicon size (bp)** |
| --- | --- | --- | --- | --- |
|  | **Spanning the BSJ**^1^ | **Used in pre-amplification** | **Used in semi-nested real-time PCR** |  |
| circ-BCL2L12-1 | CTGGCCTCGGACCAG | CTCACCACGCCTAAGGAAGG | AAGGAAGGCAGCTAGGACC | 86 |
| circ-BCL2L12-15 |  |  |  |  |
| circ-BCL2L12-16 |  |  |  |  |
| circ-BCL2L12-2b | GCCTGGCAGGCTCTG | CAGCCTCACCACGCCTAAG | CTCACCACGCCTAAGGAAGG | 77 |
| circ-BCL2L12-21 |  |  |  |  |
| circ-BCL2L12-22 |  |  |  |  |
| circ-BCL2L12-3 | CGGCTTTTGGGTCTGTTGG | GGGTCCTAGCTGCCTTCCTT | CTTAGGCGTGGTGAGGCTG | 85 |
| circ-BCL2L12-4 | ATAGGGGCGGGGCAGA | CTCCTGTTCCAACTCCACCTAG | CACCTAGCCCTGCCCAAG | 128 |
| circ-BCL2L12-5 | GGCCGGGCTCTAAACCA | GGGTCTCCTGTTCCAACTCCA | CAACTCCACCTAGAAGCCCTG | 159 |
| circ-BCL2L12-6 | TGGGAGTGCGCAGGC | GCCGCCTCCTCCACTC | CCACTCAACTTGCGGTCTCC | 77 |
| circ-BCL2L12-8 | CACTGAACTCGTACAAACTTTATTTTTGGC | TGTTTCACCCGTGGACTTGA | ACTTGCCATTGGACTGAGCTCT | 165 |
| circ-BCL2L12-10b | TTGCGGTCGCGGGGT | GAAGGAAGCCATACTGCGGA | CTGCTGGAGGAGGAGGCA | 61 |
| circ-BCL2L12-11 | GGAGGAGGAGGCGGTG | CTCACCACGCCTAAGGAAGG | AAGGAAGGCAGCTAGGACC | 101 |
| circ-BCL2L12-13 |  |  |  |  |
| circ-BCL2L12-12 | GGGCCCCACACGAAGA | CTCCTGTTCCAACTCCACCTAG | TGTTTCACCCGTGGACTTGA | 100 |
| circ-BCL2L12-14 | CATGCCCCGGACCAG | CTCACCACGCCTAAGGAAGG | AAGGAAGGCAGCTAGGACC | 86 |
| circ-BCL2L12-17b | CCTGGCCCGGACCAG | CTCTTCTTGGGCAGGGCTT | CAGCCTCACCACGCCTAAG | 102 |
| circ-BCL2L12-18 | GGGTCTAAAGGAGGACCTCTCT | CTTAGGCGTGGTGAGGCTG | CTCCTGTTCCAACTCCACCTAG | 168 |
| circ-BCL2L12-19 | CCATGGAGGCACAAAGCATAGA | TTCCTGAGCCGCCTTCG | CCCTCGGCCTTGCTCTCT | 90 |
| circ-BCL2L12-23 | CAGAGCCTGCTCCTCCA | CGGCTGGAACAGCTGGTC | CAGCTGAAATCTCCGCCCAG | 102 |
| circ-BCL2L12-24 | CCAGCTCTTCAGAGCCAGATAT | CTTAGGCGTGGTGAGGCTG | CTCCTGTTCCAACTCCACCTAG | 133 |
| circ-BCL2L12-25 | GAGCCCCTCTGAAGAGCT | CAGCCTCACCACGCCTAAG | AAGGAAGGCAGCTAGGACC | 61 |
| circ-BCL2L12-26 | TGTAGCCGGGTCTCCTGTTC | GTAGCTGGGCCAGGCTCTAA | AGAGAGCAAGGCCGAGG | 144 |
| circ-BCL2L12-27a | AAAGAGAGGACAGGACGGTTCT | CCCTCGGCCTTGCTCTCT | TCCGCCCCTGCTATGGTTTA | 164 |
| circ-BCL2L12-28 | AGAAACATTATCCAAAGAGACTGCTTGC | CGAAGGCGGCTCAGGAA | CTCTTCTTGGGCAGGGCTT | 91 |
| circ-BCL2L12-29 | GGGCAGGGACATGAGGT | TGTTTCACCCGTGGACTTGA | ACTTGCCATTGGACTGAGCTCT | 64 |
| circ-BCL2L12-30 | TAACCAGAAGCCCTGCCCAA | GCATAGAAGTCTGGAGTAGCTGG | GTAGCTGGGCCAGGCTCTAA | 160 |
| circ-BCL2L12-31 | CTTGGGCAGGGCGGG | CCAGCCCAGAATTACAGGGTC | GAAGGAAGCCATACTGCGGA | 94 |
| circ-BCL2L12-32 | ACCATAGCAGCTTCTGAGAAAGAGC | AGGGCATCCTGGCTGTTTCA | TGTTTCACCCGTGGACTTGA | 62 |
| circ-BCL2L12-33 | TGAAAATAACTAGCCACCGCACC | GGGAGGTAGAGGTTGTGGTGAG | CAAGGGCGAAACTCCGTCTC | 81 |
| circ-BCL2L12-34 | GTGCTAGGATTACAGGCGTGAG | CTTGGCAAGGACAGGAGTTG | ACTGGGATCAGAAGCTGGATCT | 119 |
| circ-BCL2L12-35a | CAACATGGTGAAACCCCATTTCTACTA | CAACTACCTTGGCAGGTGGC | CGGAGTCTCGCAATTCTCCT | 114 |
| circ-BCL2L12-36 | TCGCAATTCTCCTGACCATCC | GGAGGCAGAAGTCATTAACCAGAAG | GCATCTGTCCCACTCCTTGG | 103 |
| circ-BCL2L12-37 | GGCTGGGCCCAGCTA | GACCCTGTAATTCTGGGCTGG | GACCAGCTGTTCCAGCCG | 62 |
| circ-BCL2L12-38 | AGTAGCTGGGCCTTCTGGTTA | CAGCTGAAATCTCCGCCCAG | CCAGCCCAGAATTACAGGGTC | 112 |
| circ-BCL2L12-39 | AGCTGGGCCATTCCGC | CTGCTGGAGGAGGAGGCA | GGAGGCAGAAGTCATTAACCAGAAG | 82 |
| circ-BCL2L12-40 | GGCTGGGACAGCTGGT | TCCGCAGTATGGCTTCCTTC | GGCTTCCTTCTCTGTCGATGG | 169 |
| circ-BCL2L12-41 | GGCGGAGATGGGGTCTT | GGGTACGCTAATACTGTGAGCGT | CGGGGTAGCTCATGCCTGTA | 113 |
| circ-BCL2L12-42 | ACTCAGGGCTGCGGAG | GTCATCCCGGCTACAGAACA | GCGAAAGAGTCGGAGGACAG | 121 |
| circ-BCL2L12-43 | ACTGAGCTCTTTCTCAGAAGTCATTAACC | GTCATCCCGGCTACAGAACA | GCGAAAGAGTCGGAGGACAG | 93 |
| circ-BCL2L12-44 | TGGTGGTTGCCCTGCG | ACGTGCTCCACGCTGAGTC | GTCATCCCGGCTACAGAACA | 86 |
| circ-BCL2L12-46 | TTGGGAGGCTGAGGTGTC | TGTTTCACCCGTGGACTTGA | ACTTGCCATTGGACTGAGCTCT | 62 |
| circ-BCL2L12-47b | GAACTCGTACCCGGGGAAG | TTAACCAGAAGCTGGCCTCG | CTGTCCTCCGACTCTTTCGC | 113 |
| circ-BCL2L12-92 | AGCAGGAGGAGGAAAAGCAC | TGTTTCACCCGTGGACTTGA | ACTTGCCATTGGACTGAGCTCT | 91 |

^1^Primers spanning BSJ were used both in the pre-amplification and the semi-nested real-time PCR; they can be either forward or reverse.

**Table S3** The sequences of the siRNA duplexes used to target *BCL2L12* circRNAs, along with the scrambled sequence used as a negative control

| **Target** | **Strand direction** | **Sequence**^1^ **(5**′🡪**3**′**)** |
| --- | --- | --- |
| circ-BCL2L12-29 | Sense | CCUCAUGUCCCUGCCCAAG(TT) |
|  | Antisense | CUUGGGCAGGGACAUGAGG(TG) |
| circ-BCL2L12-92 | Sense | GCUUUUCCUCCUCCUGCUA(GG) |
|  | Antisense | UAGCAGGAGGAGGAAAAGC(AC) |
| - (Scrambled sequence) | Sense | GCCAUCAACGAUAAGUGAAG |
|  | Antisense | UUCACUUAUCGUUGAUGGCUU |

^1^The parentheses indicate dideoxynucleotide overhangs.

**Table S4** Pre-amplification and real-time qPCR primer pairs used for the relative quantification of *BCL2L12* mRNA and circ-BCL2L12-92, after siRNA transfection in HCT 116 cells

|  | **Amplified molecule** | **Direction** | **Sequence (5΄🡪3΄)** | **T_a_**^1^ **(^o^C)** |
| --- | --- | --- | --- | --- |
| **Pre-amplification** | circ-BCL2L12-92 | Forward | TTCGTGTGCTTTCCTCCTCCT | 50 |
|  |  | Reverse | TCAAGTCCACGGGTGAAACA |  |
|  | ciRS-7 | Forward | TCTGCTCGTCTTCCAACATC | 48 |
|  |  | Reverse | GCTCAGGATTATCTGGAAGACC |  |
| **Real-time qPCR** | circ-BCL2L12-92 | Forward | TTCGTGTGCTTTCCTCCTCCT | 60 |
|  |  | Reverse | GAAACAGCCAGGATGCCCTC |  |
|  | ciRS-7 | Forward | TACCCAGTCTTCCATCAACTGG | 60 |
|  |  | Reverse | ACACAGGTGCCATCGGAAAC |  |
|  | *BCL2L12* mRNA | Forward | TTCCTGAGCCGCCTTCG | 60 |
|  |  | Reverse | GCATAGAAGTCTGGAGTAGCTGG |  |
|  | *GAPDH* mRNA | Forward | TCAAGGCTGAGAACGGGAA | 60 |
|  |  | Reverse | CGCCCCACTTGATTTTGGAG |  |
|  | *ACTB* and *ACTG1* mRNAs | Forward | CACCATTGGCAATGAGCGGTT | 60 |
|  |  | Reverse | AGGTCTTTGCGGATGTCCACGT |  |

^1^Annealing temperature.

**Supplementary Figures**


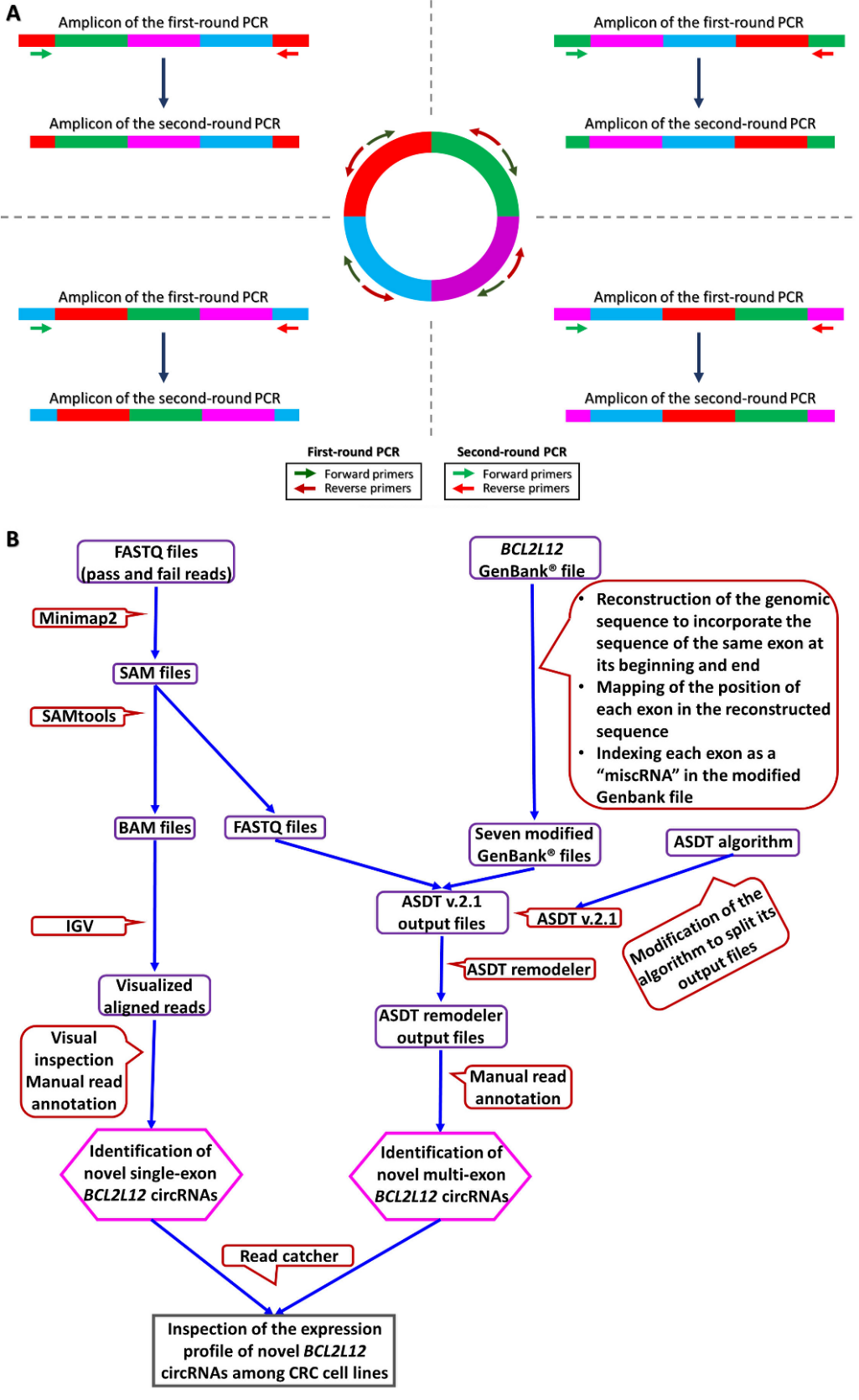


**Fig. S1**  The concept of amplifying and sequencing cDNAs corresponding to circRNAs, followed by nanopore sequencing data analysis, to reveal the identity of novel *BCL2L12* circRNAs. **(A)** Schematic representation of an example of two consecutive PCRs amplifying *BCL2L12* circRNAs consisting of 4 exons, with the use of two distinct pairs of divergent PCR primers. For simplicity, only one such *BCL2L12* circRNA is depicted. **(B)** A flowchart stepwise presenting the bioinformatics pipeline developed and used in this research study is shown.


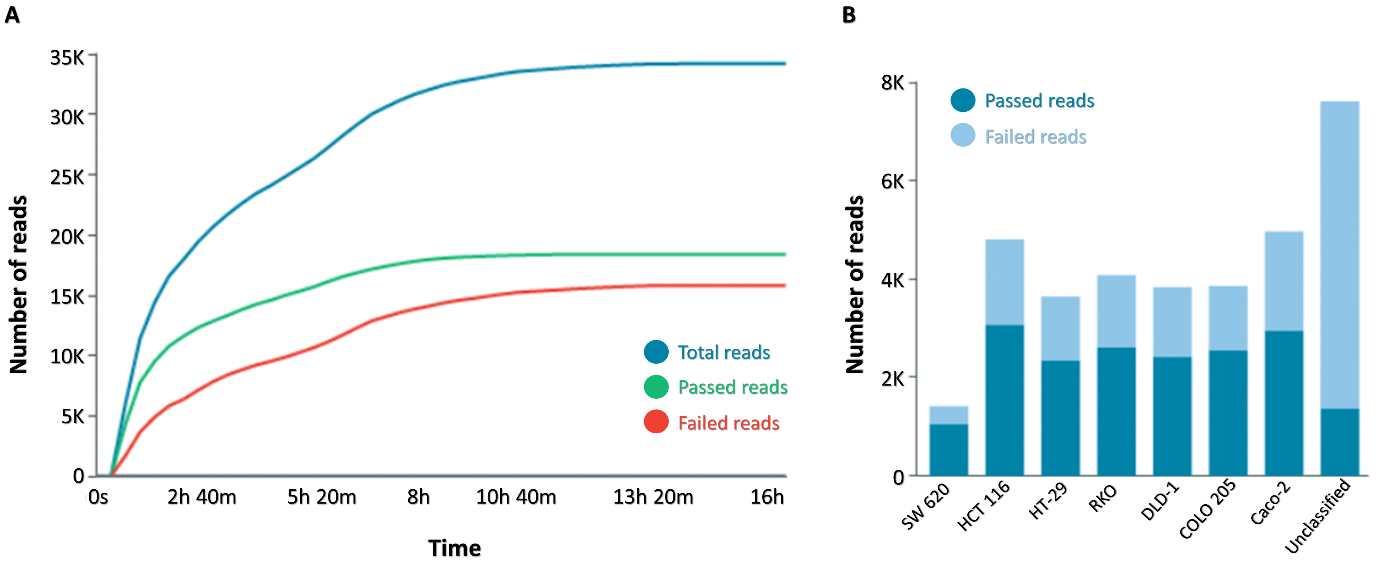


**Fig. S2**  Metrics of the nanopore sequencing run. **(A)** The cumulative number of sequencing reads is plotted against the accrued time of the nanopore sequencing experiment. **(B)** This plot shows the number of passed and failed sequencing reads per CRC cell line, as well as the number of reads that could not be assigned to any cell line due to sequencing error(s) in the attached barcode of each read.


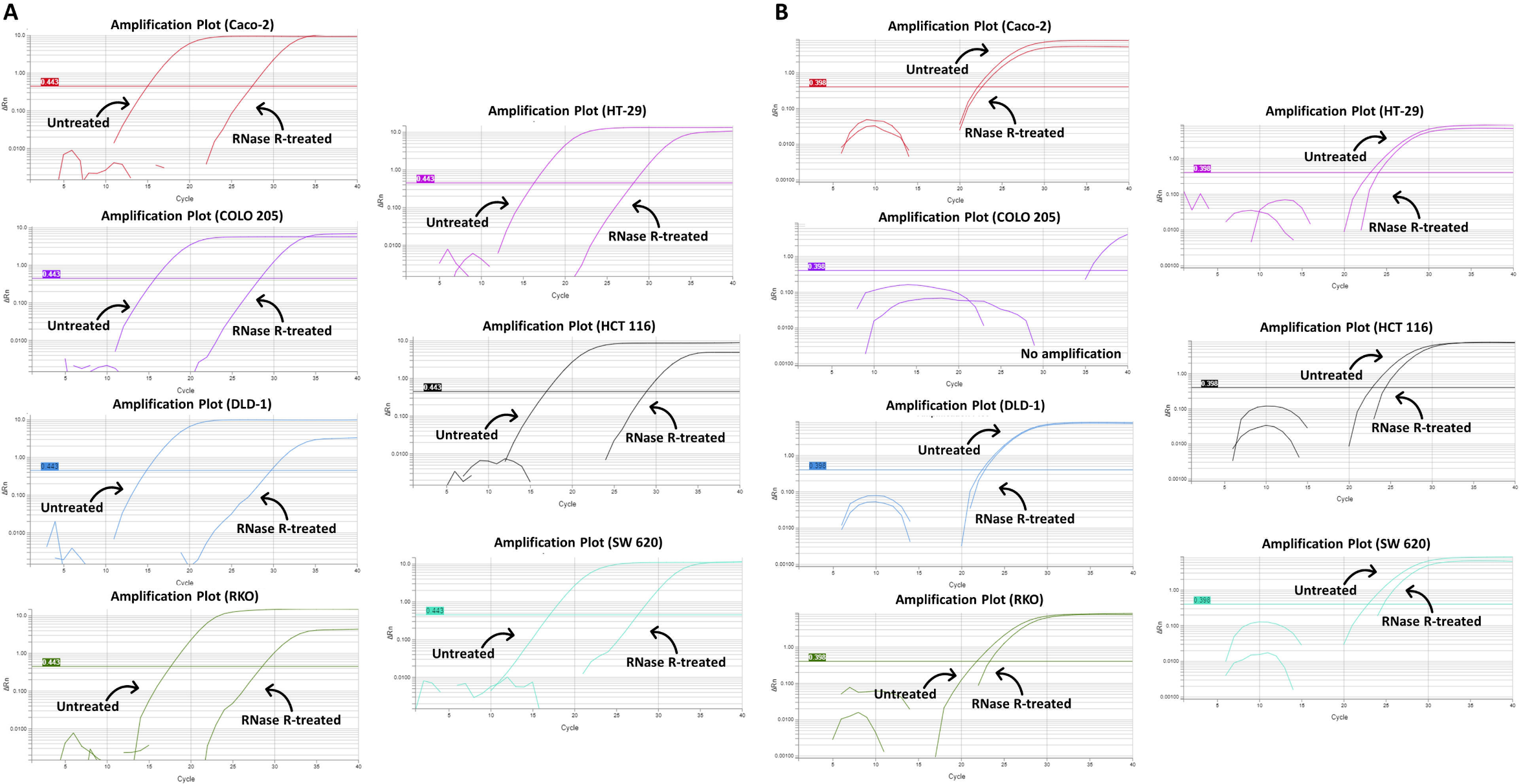


**Fig. S3**  Amplification plots of *GAPDH* mRNA **(A)** and ciRS-7 **(B)** before and after RNase R treatment of the total RNA extracts of the 7 CRC cell lines. ciRS-7 was not detected in the COLO 205 cell line.


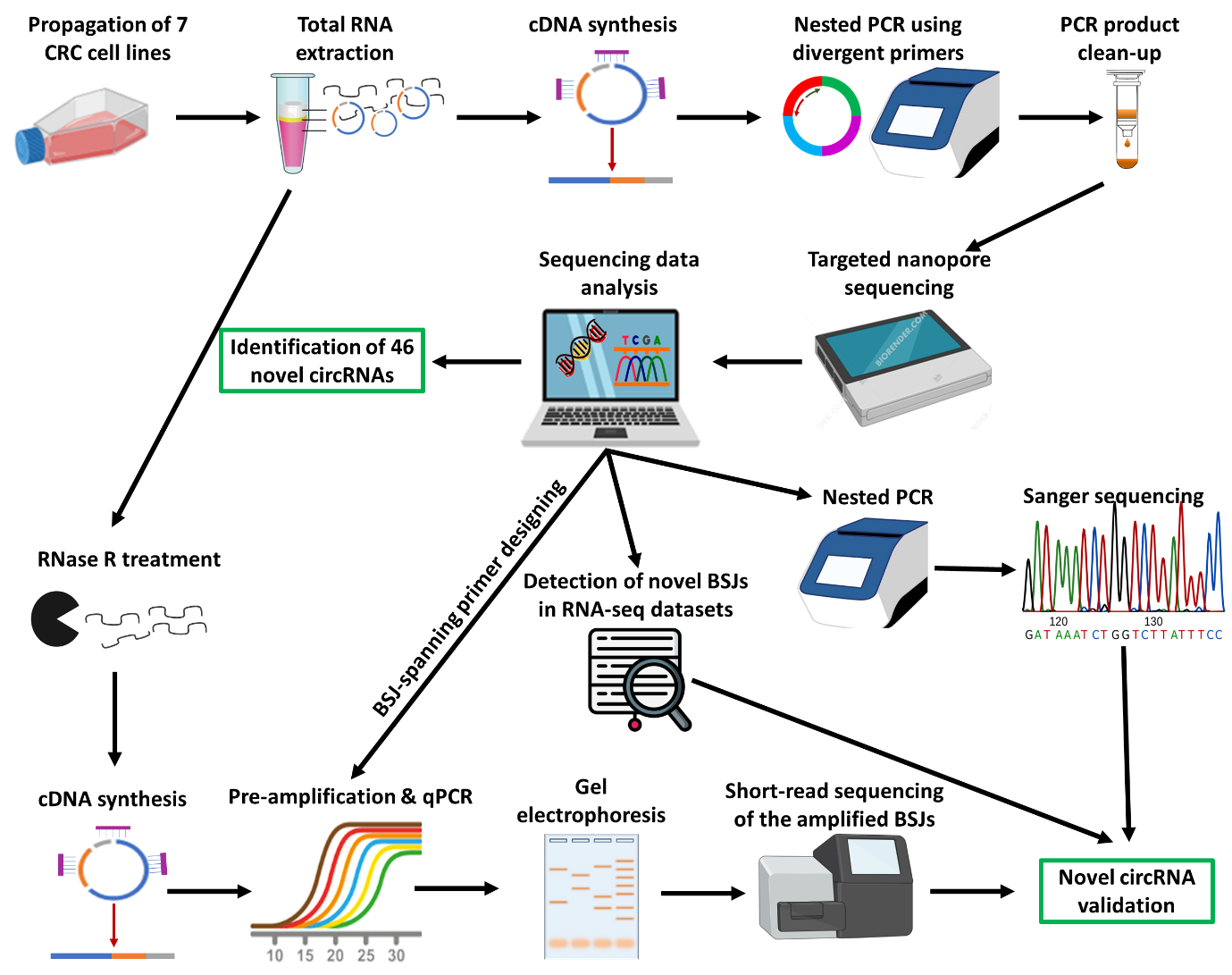


**Fig. S4** Illustration of our experimental workflow for the identification of novel *BCL2L12* circRNAs.


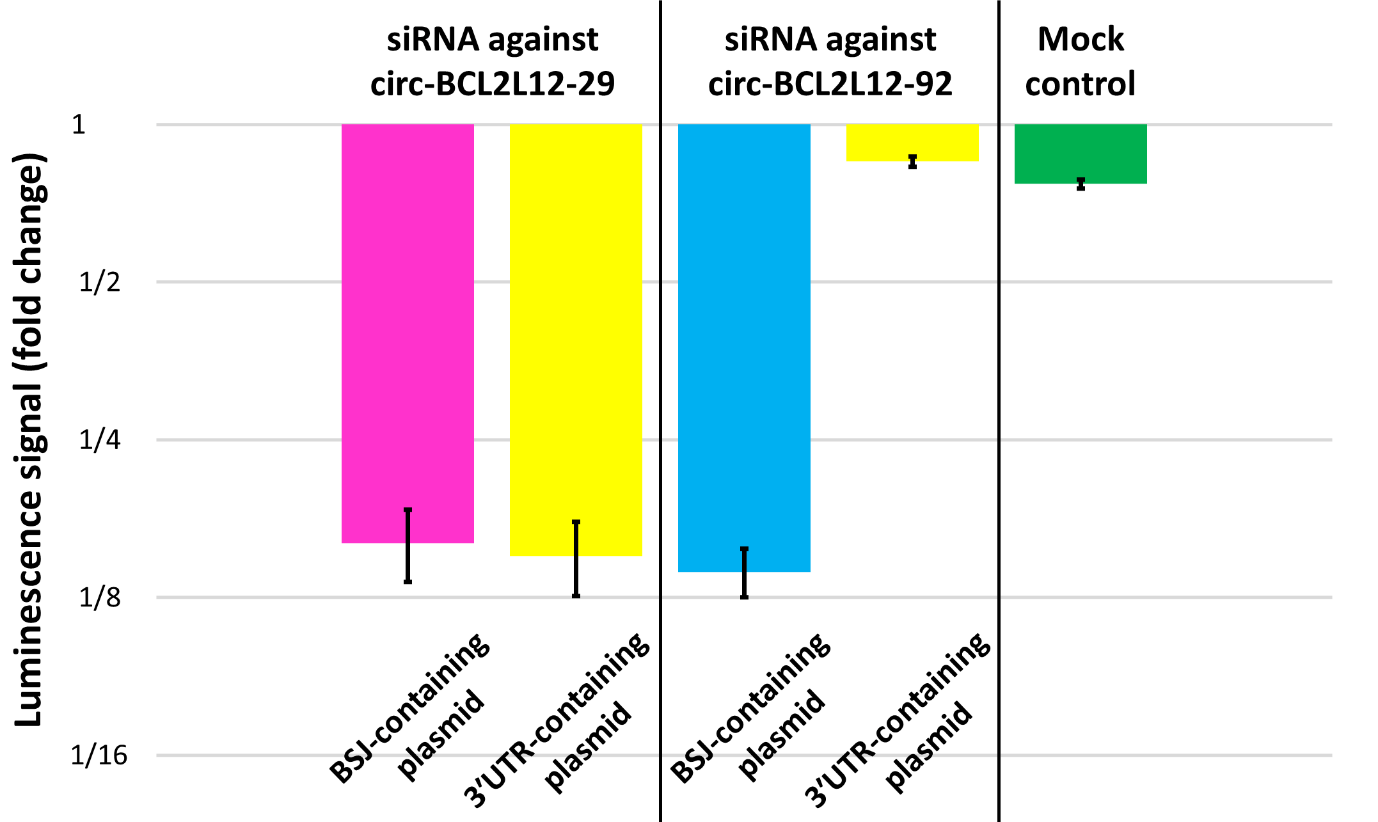


**Fig.** **S5** Presentation of the dual-luciferase reporter assay results. Normalized ratios were obtained by calculating the Renilla to firefly luminescence signal ratio, and then by dividing this ratio with the respective one calculated for the negative control (co-transfected HCT 116 cells with plasmid and scrambled siRNA sequence). Each bar depicts the mean expression along with its standard error, obtained by 3 technical replicates in each case.


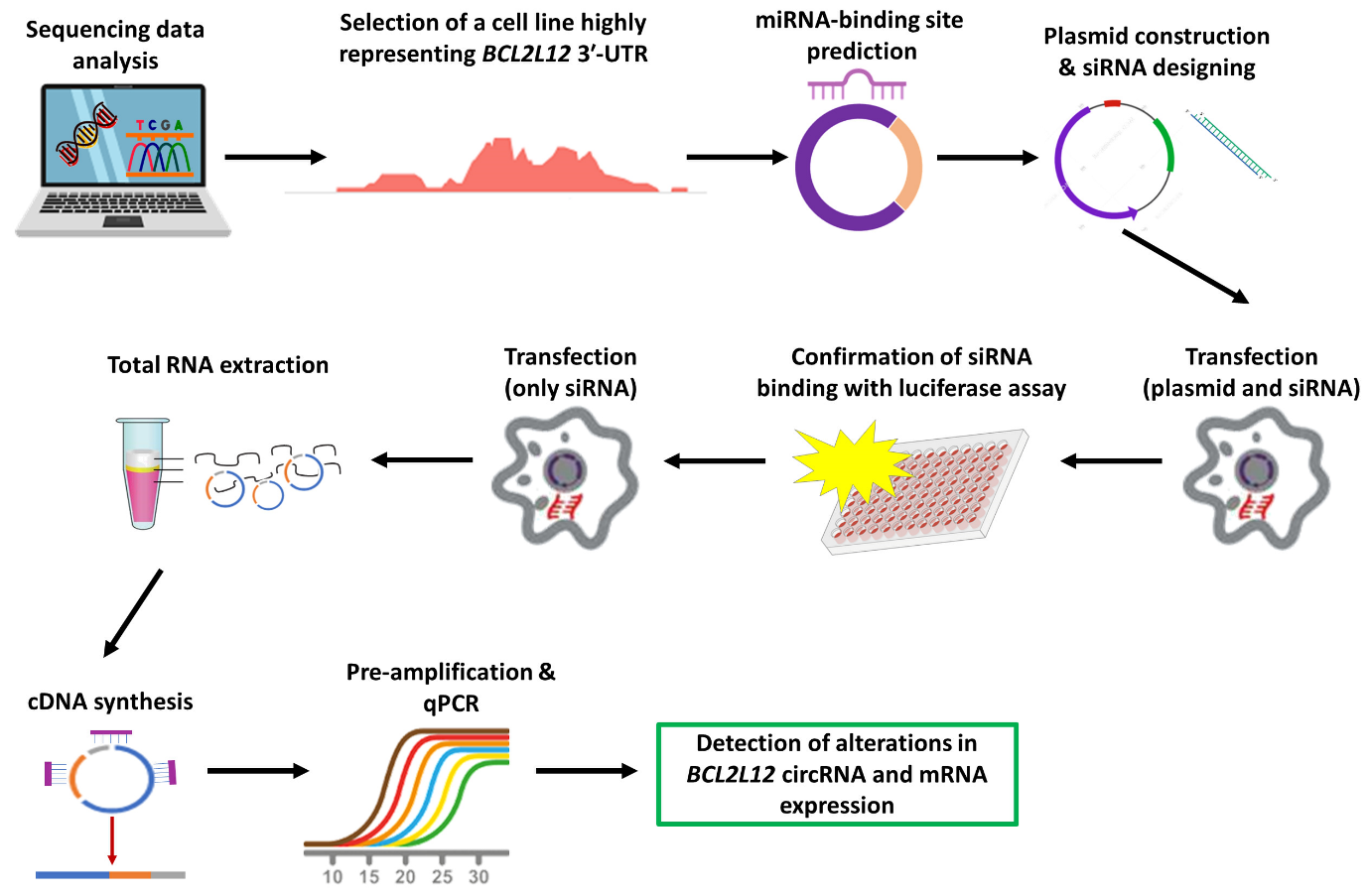


**Fig. S6**  Illustration of the workflow followed to explore the putative effect of the downregulation of selected novel circRNAs on *BCL2L12* mRNA expression.
